# Type I Interferon Production Induced by *Streptococcus pyogenes*-Derived Nucleic Acids Is Required for Host Protection

**DOI:** 10.1371/journal.ppat.1001345

**Published:** 2011-05-19

**Authors:** Nina Gratz, Harald Hartweger, Ulrich Matt, Franz Kratochvill, Marton Janos, Stefanie Sigel, Barbara Drobits, Xiao-Dong Li, Sylvia Knapp, Pavel Kovarik

**Affiliations:** 1 Max F. Perutz Laboratories, Department of Microbiology, Immunobiology and Genetics, University of Vienna, Vienna, Austria; 2 CEMM, Research Center for Molecular Medicine of the Austrian Academy of Sciences and Department of Internal Medicine I, Division of Infectious Diseases and Tropical Medicine, Medical University of Vienna, Vienna, Austria; 3 Institute for Cancer Research, Department of Internal Medicine I, Medical University of Vienna, Vienna, Austria; 4 Department of Molecular Biology, UT Southwestern Medical Center, Dallas, Texas, United States of America; Dartmouth Medical School, United States of America

## Abstract

*Streptococcus pyogenes* is a Gram-positive human pathogen that is recognized by yet unknown pattern recognition receptors (PRRs). Engagement of these receptor molecules during infection with *S. pyogenes*, a largely extracellular bacterium with limited capacity for intracellular survival, causes innate immune cells to produce inflammatory mediators such as TNF, but also type I interferon (IFN). Here we show that signaling elicited by type I IFNs is required for successful defense of mice against lethal subcutaneous cellulitis caused by *S. pyogenes*. Type I IFN signaling was accompanied with reduced neutrophil recruitment to the site of infection. Mechanistic analysis revealed that macrophages and conventional dendritic cells (cDCs) employ different signaling pathways leading to IFN-beta production. Macrophages required IRF3, STING, TBK1 and partially MyD88, whereas in cDCs the IFN-beta production was fully dependent on IRF5 and MyD88. Furthermore, IFN-beta production by macrophages was dependent on the endosomal delivery of streptococcal DNA, while in cDCs streptococcal RNA was identified as the IFN-beta inducer. Despite a role of MyD88 in both cell types, the known IFN-inducing TLRs were individually not required for generation of the IFN-beta response. These results demonstrate that the innate immune system employs several strategies to efficiently recognize *S. pyogenes*, a pathogenic bacterium that succeeded in avoiding recognition by the standard arsenal of TLRs.

## Author Summary

*Streptococcus pyogenes* is an important human pathogen that causes a broad range of diseases. The bacterium colonizes the throat and the skin where it can evoke usually mild illness such as strep throat or scarlet fever. Systemic infections with *S. pyogenes* are less frequent but can develop into life-threatening diseases such as necrotizing fasciitis and streptococcal toxic shock syndrome. The immune system launches a usually successful response that is initiated by a so far not understood recognition of this pathogen by the cells of the innate immune system. These cells produce upon infection a variety of cytokines that orchestrate a full blown protective response. Among these cytokines, type I interferons play a critical role as demonstrated by our study. We further show that IFN-beta, the key type I interferon, is produced only after macrophages and dendritic cells have taken up the pathogen and liberated the bacterial nucleic acids for recognition in the intracellular vesicles. Importantly, macrophages and dendritic cells recognize different nucleic acids and employ different signaling pathways to respond. Our data suggest that the innate immune system employs several strategies to efficiently recognize *S. pyogenes*, a pathogenic bacterium that succeeded in avoiding recognition by the standard recognition mechanisms.

## Introduction

*Streptococcus pyogenes*, also known as Group A streptococcus (GAS), is a Gram-positive human pathogen causing an exceptionally broad range of infectious diseases [1]. The bacterium can evoke mild illnesses such as pharyngitis or scarlet fever. Systemic infections with *S. pyogenes* can develop into life-threatening diseases such as necrotizing fasciitis and streptococcal toxic shock syndrome that occur in ∼10000 cases in the US annually resulting in ∼1500 deaths [2]. The wide spectrum of *S. pyogenes*-related diseases arises from diversity in the genetic inventory of both the host and the pathogen [3], [4], [5], [6]. Genetic linkage analysis of mouse strains differing in their susceptibility to *S. pyogenes* infections demonstrated importance of the innate immune system [7]. Animal studies confirmed an essential role of macrophages and dendritic cells (DCs) in protection against *S. pyogenes* infections [8], [9].

Innate immune cells initiate the immune response by production of inflammatory mediators which results from the recognition of the pathogen-associated molecular patterns (PAMPs) by pattern recognition receptors (PRRs) [10]. Recognition of bacteria is achieved by membrane-bound PRRs of the TLR family and by several classes of cytosolic PRRs. In mice, 12 TLRs are known [10], [11]. They all signal through the adaptor MyD88, except for TLR3, which employs the TRIF. TRIF is also used by TLR4, together with MyD88. Further downstream events include the activation of MAPKs and transcription factors of the NF-κB and IRF families. TLR3, TLR7, TLR8 and TLR9 are localized in the endosomal compartments whereas the remaining TLRs reside at the cell membrane [12]. Signaling from the cell membrane-bound TLRs leads to the production of NF-κB -driven cytokines (e.g. TNF, IL-6) while signaling from the endosomal TLRs triggers the induction of IRF-driven interferon beta (IFN-β) and NF-κB-driven cytokines. The initially cell surface-localized TLR2 and TLR4 turn into IFN-β inducers after ligand-triggered internalization [13], [14]. Bacterial products that reach the cytosol can be recognized in MyD88-independent ways by cytosolic PRRs including nucleotide-binding and oligomerization domain (NOD)-like receptors (NLRs), retinoic acid-inducible gene I (RIG-I)-like receptors (RLRs) and the DNA-sensors DAI, AIM2 and LRRFIP1 [10], [15]. NLRs and AIM2 cause inflammasome-dependent IL-1β release. In addition, the NLR members NOD1 and NOD2 activate NF-κB -driven cytokine production [16]. RLRs, DAI and LRRFIP1 are involved in type I IFN production in response to bacteria-derived nucleic acids.

The recognition of *S. pyogenes* is not understood. Although *S. pyogenes* cannot divide in infected cells, phagosomal escape and a limited cytosolic survival have been reported so that both extra- and intracellular recognition are possible [17], [18]. Studies by us and others demonstrated that murine bone marrow-derived macrophages (BMDMs) and conventional DCs (cDCs) responded to *S. pyogenes* by a TLR2-, TLR4- and TLR9-independent production of TNF and IL-6 [19], [20]. The lack of requirement for TLR2 is surprising since *S. pyogenes* contains the TLR2 ligand lipoteichoic acid [21]. The TNF and IL-6 production was strictly dependent on MyD88 that was also needed for survival of *S. pyogenes*-infected mice [19], [22]. Another route of *S. pyogenes* recognition employs the activation of the Nlrp3 inflammasome by a so far unknown ligand [23]. The inflammasome-induced IL-1 could not account for the MyD88-dependent induction of IL-6 and TNF since IL1-R1^-/-^ BMDMs were fully responsive to *S. pyogenes* [19]. Our previous study showed that *S. pyogenes* induced IFN-β in BMDMs, which was the first evidence for a Gram-positive bacterium with mostly extracellular life cycle to do so [19]. Subsequently, the extracellular Group B Streptococcus (GBS) was also shown to induce IFN-β in BMDMs and cDCs [24], [25]. The mechanism of IFN-β induction by *S. pyogenes* and the role of type I IFN signaling in a valid infection model remained to be elucidated. Depending on the pathogen, type I IFN signaling can have protective or deleterious effects in infection models [26]. Here we show that mice deficient in type I IFN signaling are susceptible to invasive *S. pyogenes* infection. cDCs required MyD88 and IRF5 for IFN-β production, while BMDMs possessed MyD88-dependent and independent pathways that however both required IRF3. In both cell types, IFN-β was induced in the absence of the known IFN-β -inducing PRRs TLR3, TLR7, TLR9, NOD1 or NOD2. Analysis of bacterial components indicated that *S. pyogenes*-derived RNA was the IFN-β inducer in cDCs whereas bacterial DNA was the inducer in BMDMs. Our data also propose that induction of IFN-β by *S. pyogenes* and GBS is achieved by similar PAMPs but different sensing mechanisms.

## Results

### Type I IFN signaling is required for host protection against *S. pyogenes* infection in lethal cellulitis

**Figure 1:**
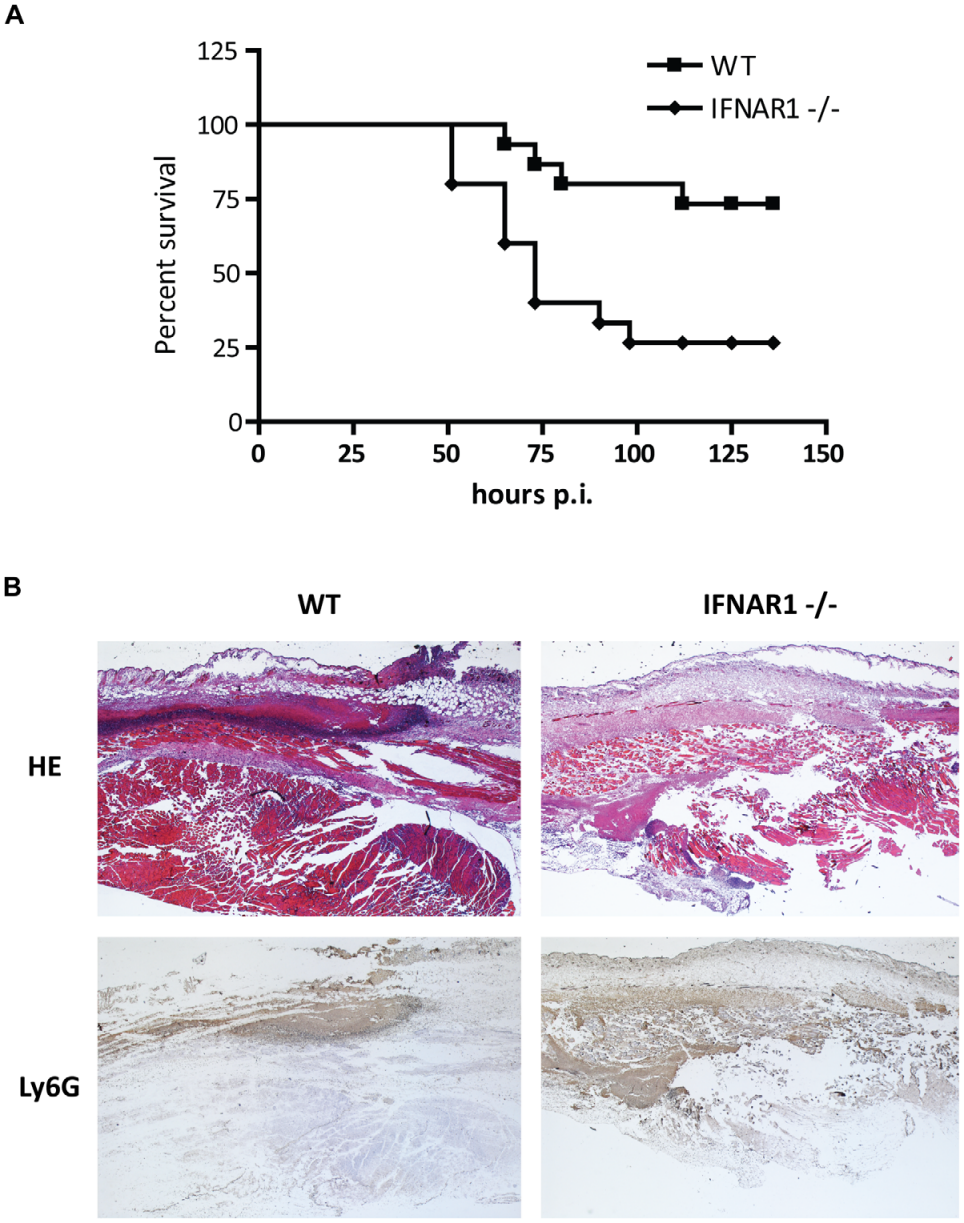
Type I IFN signaling is needed for host defense against *S. pyogenes* and control of neutrophil recruitment. (**A**) Kaplan-Meier survival curves of C57BL/6 and IFNAR1^-/-^ mice (15 mice per genotype) after subcutaneous infection with 3×10^8^ CFU of *S. pyogenes*. Survival was monitored for 6 days. Significance: **  = P value<0.01. (**B**) Representative HE-stained (upper panel) and Ly6G-stained (lower panel) sections of flanks from *S. pyogenes*-infected control (WT) and IFNAR1^-/-^ mice 48 h after infection are shown. (Magnification: ×25). Note exaggerated tissue destruction (upper panel) and increased neutrophil (lower panel) recruitment in samples from IFNAR1^-/-^ mice.

Recent studies revealed that, depending on the pathogen, type I IFNs may be beneficial or harmful to the host [25], [27], [28], [29]. To test how the *S. pyogenes*-induced type I IFNs contribute to host defense, we subcutaneously infected type I IFN receptor 1-deficient (IFNAR1^-/-^) and control (WT) mice and monitored survival over 6 days. This infection model resembles human skin infections with *S. pyogenes* which give rise to cellulitis and may develop into streptococcal toxic shock syndrome [30]. IFNAR1^-/-^ mice displayed 75% lethality rate whereas only 25% of WT controls died demonstrating essential contribution of type I IFNs to the host defense against *S. pyogenes* (Fig. 1A). Inspection of animals 2 days post challenge revealed that neutrophils were more abundant at the site of infection in the IFNAR1^-/-^ mice indicating that type I IFNs negatively regulated neutrophil recruitment (Fig. 1B). Tissue destruction was more advanced in the wound of IFNAR1^-/-^ mice (Fig. 1B) in accordance with the tissue-eroding activity of enzymes released from neutrophils. These findings suggest that excessive neutrophil activity at the site of infection negatively influences the outcome of infection.

### Macrophages and dendritic cells employ different mechanisms for IFN-β induction by *S. pyogenes*

**Figure 2:**
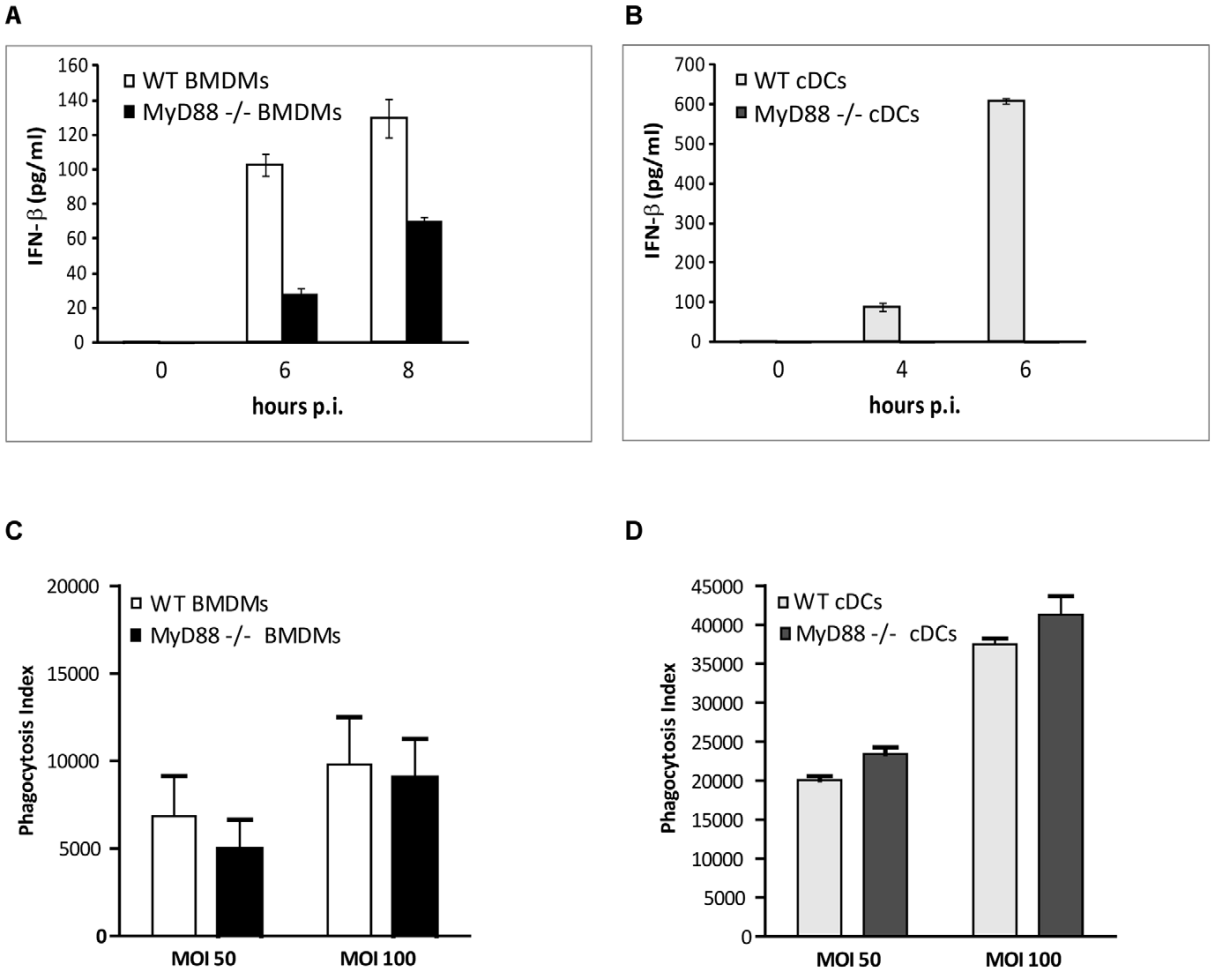
MyD88 is involved in IFN-β induction by *S. pyogenes* in BMDMs and cDCs. (**A, B**) IFN-β production in BMDMs is partially (A) and in cDCs completely (B) dependent on MyD88. Cells from wild-type (WT) and MyD88^-/-^ mice were infected with *S. pyogenes* (MOI = 100) or left untreated. After indicated periods, supernatants were collected and IFN-β release was measured by ELISA. Values represent mean ± SD; n = 3. (**C, D**) Control (WT) and MyD88^-/-^ BMDMs (C) and cDCs (D) were infected with CFSE-labeled *S. pyogenes* (MOI = 50 and MOI = 100) for 45 min. Uptake of bacteria was determined using flow cytometry. Results are expressed as phagocytosis index, defined as the percentage of cells with internalized *S. pyogenes*, multiplied by the mean fluorescent intensity. Statistical analysis (see Material and Methods) did not reveal significant differences between WT and MyD88^-/-^ cells.

Macrophages (BMDMs) and cDCs are required for protection of mice against *S. pyogenes* infections and for preventing dissemination of bacteria from the primary subcutaneous inoculation site [8], [9]. Both cell types respond to *S. pyogenes* infection by a robust TNF and IL-6 production that is MyD88-dependent and involves a so far unidentified receptor different from the common receptors for bacterial components i.e. TLR2, TLR4 and TLR9 [19], [20]. Both cell types were also reported to induce IFN-β although the precise mechanisms remained unknown [19], [25]. We directly compared the requirement for MyD88 in *S. pyogenes*-induced IFN-β production by infecting BMDMs and cDCs derived from MyD88^-/-^ and WT mice. IFN-β amounts were measured in supernatants collected 4 and 6 hrs after infection of cDCs, and 6 and 8 h after infection of BMDMs since the accumulation of the cytokine in the supernatants took consistently longer in BMDMs as compared to cDCs (Fig. S1A). No further increase of IFN-β was observed 16 h after infection (data not shown). The IFN-β induction was dose-dependent (Fig. S1B). In BMDMs the amount of IFN-β was reduced by 50% in the absence of MyD88 while the IFN-β production in cDCs was fully MyD88-dependent (Fig. 2A, B). The lack of IFN-β induction in MyD88^-/-^ was not caused by reduced phagocytosis since the number of internalized bacteria in these cells was comparable to control cells (Fig. 2C, D). We observed that the IFN-β-driven activation of the transcription factor Stat1 was slightly higher in MyD88^-/-^ than in WT BMDMs although the Stat1 protein levels were lower in the knockout cells (Fig. S2A). We reasoned that the increased Stat1 activation despite of a reduced IFN-β production in MyD88^-/-^ cells might be caused by the inability of MyD88^-/-^ cells to activate expression of the *Socs1* gene, an inhibitor of IFN signaling. Full expression of *Socs1* requires activation of the p38 MAPK [31] that is reduced in *S. pyogenes*-infected MyD88^-/-^ cells [19]. Consistently, *Socs1* mRNA was not induced by *S. pyogenes* in MyD88^-/-^ BMDMs (Fig. S2B).

**Figure 3:**
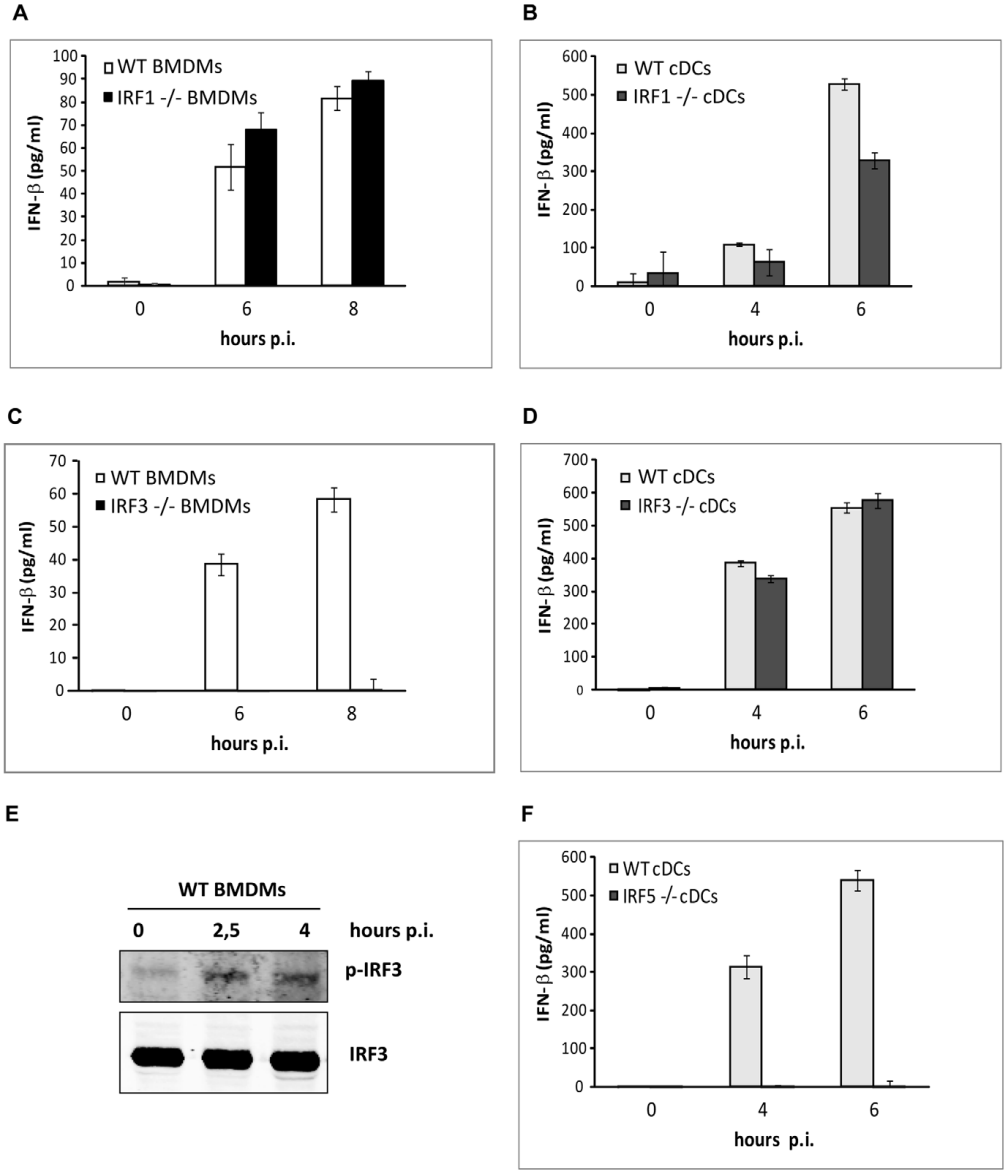
BMDMs require IRF3 while cDCs need IRF5 for IFN-β induction by *S. pyogenes*. BMDMs and cDCs from IRF1^-/-^ (**A, B**) and IRF3^-/-^ (**C, D**) mice as well as control mice (WT) were infected with *S. pyogenes* (MOI = 100) or left untreated. At indicated time points, supernatants were collected and IFN-β release was measured by ELISA. SDs (n≥3) are shown. (**E**) BMDMs were infected with *S. pyogenes* (MOI = 100). After indicated time points whole cell extracts were prepared. Activation of IRF3 was determined via Western Blot using an antibody against phosphorylated IRF3 (p-IRF3). Equal loading was confirmed by reprobing the membrane with an antibody against total IRF3. (**F**) cDCs from IRF5^-/-^ and control mice were infected with *S. pyogenes* and IFN-β production was measured as described in (A).

In cDCs, IFN-β production induced by a variety of stimuli is often regulated by the transcription factors IRF1 and IRF7 downstream of MyD88, whereas in BMDMs so far only IRF3-dependent MyD88-independent induction of IFN-β has been described [10]. Recently, IRF5 was reported to contribute to IFN-β induction by *Mycobacterium tuberculosis* in BMDMs [32]. To address the role of IRFs in *S. pyogenes*-induced IFN-β production we examined BMDMs and cDCs derived from IRF1^-/-^, IRF3^-/-^ and IRF5^-/-^ mice. IRF1 was dispensable, while IRF3 was absolutely essential for IFN-β induction in BMDMs (Fig. 3A, C). The requirement for IRF3 in BMDMs was further supported by increased phosphorylation of IRF3 in *S. pyogenes*-infected BMDMs (Fig. 3E). In contrast, IFN-β production in cDCs was partially dependent on IRF1 but not on IRF3 (Fig. 3B, D). Interestingly, IFN-β production was completely abolished in IRF5^-/-^ cDCs which is to our knowledge the first report of such dominant effect of IRF5 in this cell type (Fig. 3F). The data in cDCs are consistent with a previous study describing partial dependency of *S. pyogenes*-induced IFN-β on IRF1 [25]. In that study GBS-induced IFN-β in cDCs was completely dependent on IRF1 indicating that the two streptococcal species employ different mechanisms for IFN-β induction in cDCs. In BMDMs, the complete dependency of *S. pyogenes*-induced IFN-β on IRF3 (Fig. 3C) resembled the reported GBS-stimulated IFN-β in the same cell type [24]. However, GBS-induced IFN-β did not require MyD88 in BMDMs [24] whereas our data show that upon *S. pyogenes* infection about 50% of IFN-β depends on MyD88 (Fig. 2A). Together, the data illustrate that IFN-β production in response to *S. pyogenes* completely requires MyD88 and IRF5, with a limited contribution of IRF1 in cDCs; while in BMDMs IRF3 fully controls IFN-β generation, 50% of which is MyD88-dependent. In addition, *S. pyogenes* and GBS significantly differ in the requirement for MyD88 and IRFs in IFN-β induction, thereby further illustrating the diversity of these streptococcal pathogens.

### *S. pyogenes* induces IFN-β by using receptors that differ from PRRs typically engaged by bacterial products

**Figure 4:**
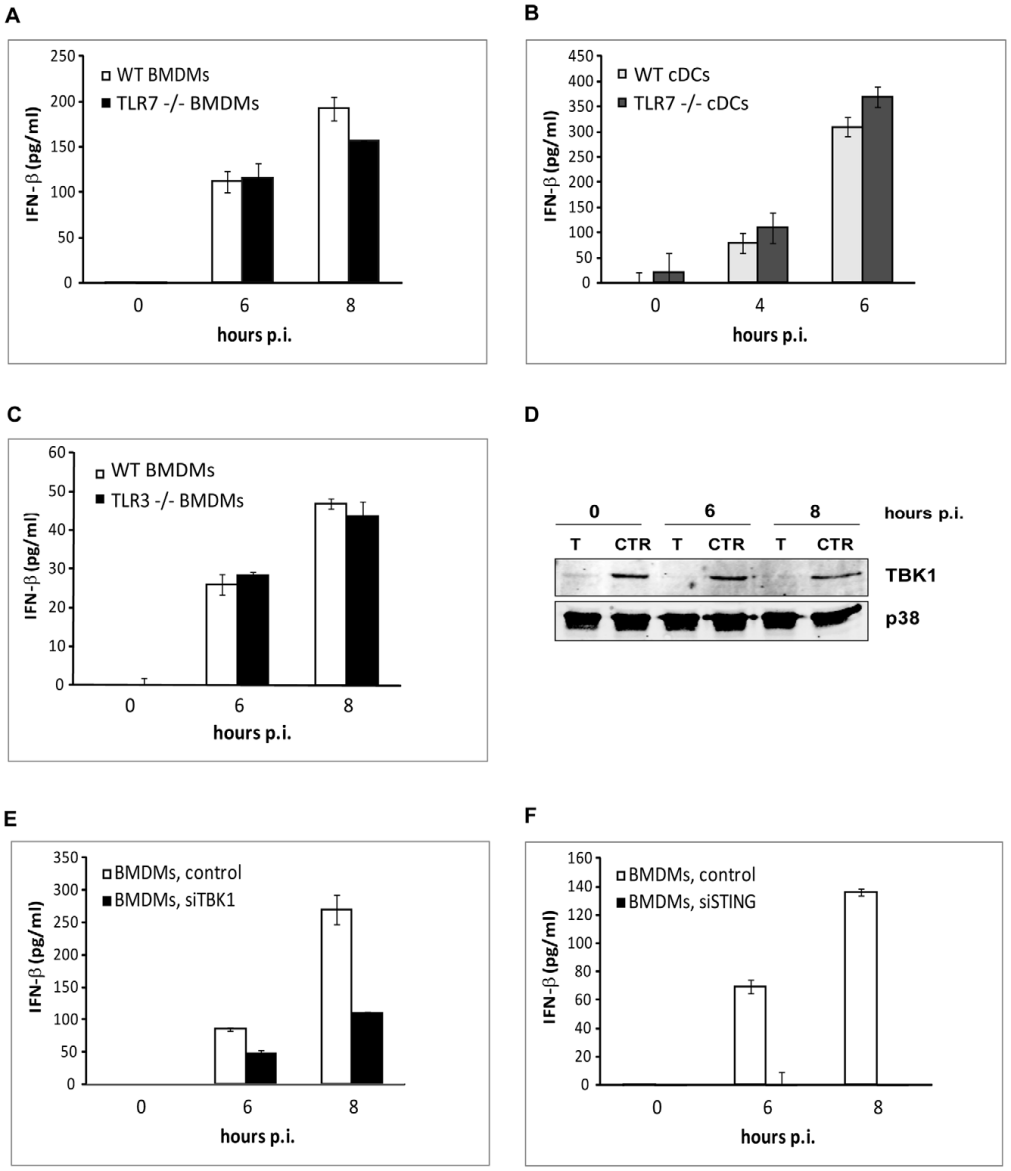
Analysis of TLR3, TLR7, TBK1 and STING in induction of IFN-β by *S. pyogenes.* BMDMs (**A**) and cDCs (**B**) from TLR7^-/-^ and control (WT) mice were infected with *S. pyogenes* (MOI = 100) for the indicated periods or left untreated. Supernatants were collected and IFN-β release was measured by ELISA. SDs (n≥3) are shown. (**C**) BMDMs from TLR3^-/-^ and control (WT) mice were infected with *S. pyogenes* and IFN-β production was measured as described in (A). (**D**) Silencing of TBK1 was monitored by Western blot analysis of extracts prepared from cells used in (E). TBK1 expression was determined using an antibody against TBK1. Equal loading was determined by re-probing the membrane with an antibody to p38 MAPK. (**E, F**) BMDMs transfected with siRNA specific for TBK1 (E), STING (F) or non-target control siRNA were infected with *S. pyogenes* and IFN-β production was measured as described in (A). SDs (n = 3) are shown.

**Figure 5:**
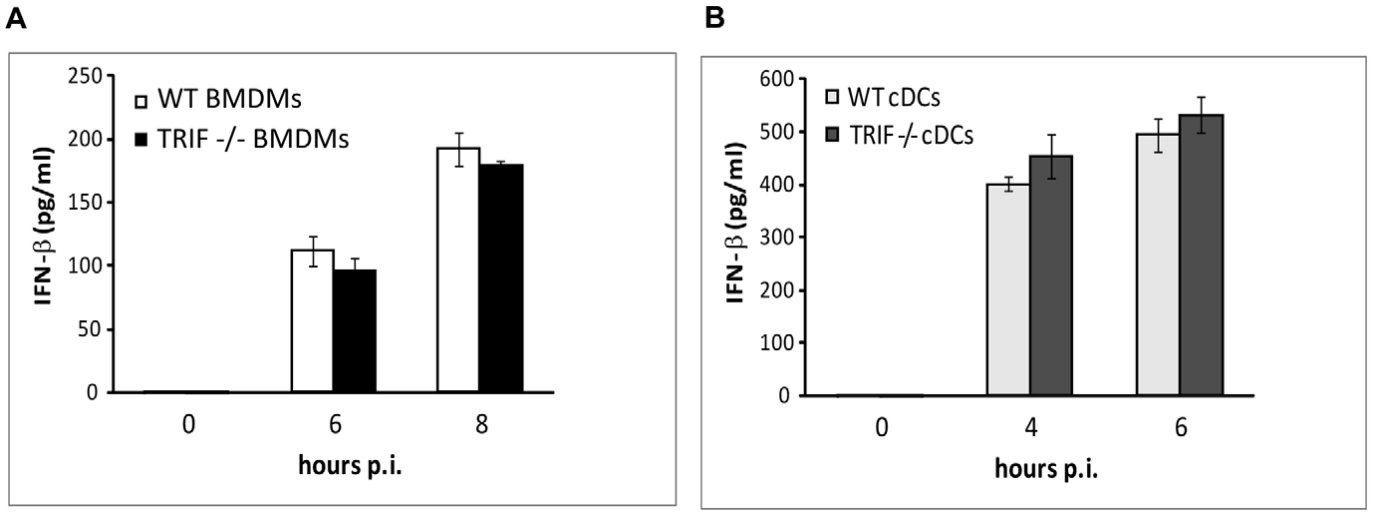
TRIF is not required for *S. pyogenes*-mediated IFN-β production. BMDMs (**A**) and cDCs (**B**) from control (WT) and TRIF^-/-^ mice were infected with *S. pyogenes* (MOI = 100). After indicated time points, supernatants were collected and IFN-β release was measured using ELISA. SDs (n≥3) are shown.

Our findings that cDCs fully and BMDMs partially require MyD88 for *S. pyogenes*-induced IFN-β production prompted us to test the involvement of the IFN-β-inducing MyD88-dependent TLR7 and the MyD88-independent TLR3. A role for TLR9 in cDCs was excluded in the study by Mancuso et al. [25]. Similarly, no requirement for TLR9 was detected in BMDMs (Fig. S3) which is in agreement with our previous data obtained using TLR2/TLR4/TLR9-triple-deficient cells [19]. TLR7^-/-^ BMDMs and cDCs infected with *S. pyogenes* did not differ in their ability to generate IFN-β when compared to WT controls (Fig. 4A, B). Similarly, deficiency in TLR3 did not result in changes of IFN-β induction in *S. pyogenes*-infected BMDMs (Fig. 4C). The role of TLR3 in cDCs has been ruled out previously [25]. Thus, neither TLR7, shown to be needed for GBS-induced IFN-β in cDCs [25], nor TLR3 could account for the recognition mechanism causing IFN-β production in *S. pyogenes*-infected cells. To further characterize pathways engaged in *S. pyogenes* recognition we examined the function of the TANK-binding kinase 1 (TBK1), a major activator of IRF3. Since IRF3 is essential for IFN-β generation by *S. pyogenes*-infected BMDMs (Fig. 3C) we silenced TBK1 in these cells using siRNA. Reduction of TBK1 protein levels to approximately 20% of the non-target siRNA control resulted in a 50% drop of IFN-β (Fig. 4D, E). Using a similar approach, we also show a requirement for the adaptor STING (Fig. 4F, fig.S4), which has been shown in a number of studies to facilitate TBK1-mediated IFN-β induction [33]. The more profound effect of silencing of STING as compared to TBK1 could reflect a more efficient silencing (perhaps due to shorter half life of the STING protein) or activation of IRF3 by a kinase other than TBK1 e.g. IKK-ε [26]. Thus, the TBK1/STING pathway was needed for *S. pyogenes*-elicited IFN-β induction in BMDMs but the involvement of TLRs known to signal via TBK1 was not detected. TBK1 can be activated in a TLR3-dependent MyD88-independent way by the adaptor TRIF [10]. However, TRIF deficiency did not result in a decreased IFN-β production in BMDMs and cDCs (Fig. 5A, B), which was consistent with the lack of any effect of TLR3 in these cells (Fig. 4C and [25]).

Recently, the cytosolic PRRs nucleotide-binding oligomerization domain (NOD) 1 and 2 were unexpectedly found to be required for IFN-β induction under certain conditions. Binding of the NOD1 ligand, a peptide derived from bacterial peptidoglycan, to NOD1 in epithelial cells resulted in a TBK1-dependent production of IFN-β [34]. In the case of NOD2, binding of virus-derived single stranded RNA (ssRNA) to NOD2 caused activation of IRF3 and induction of IFN-β [35]. NOD2 is also needed for IRF5-dependent IFN-β induction by *M. tuberculoses*-derived peptidoglycan in macrophages [32]. We excluded the involvement of NOD1 and NOD2 in *S. pyogenes*-induced IFN-β since in BMDMs from NOD1^-/-^ and NOD2^-/-^ mice the activation of Stat1 and production of IFN-β were comparable to WT cells (Fig. S5A, B).

Cumulatively, the MyD88-dependent production of IFN-β in response to *S. pyogenes* infection does not result from activation of TLR7, the TLR utilizing MyD88. Furthermore, the MyD88-independent IFN-β induction in BMDMs cannot be explained by the activation of TLR3, NOD1 or NOD2, the PRRs employing TBK1 and IRF3 signaling for IFN-β induction, despite the requirement of these two signaling components for *S. pyogenes*–stimulated IFN-β production.

### Processing of internalized *S. pyogenes* is required for IFN-β production

**Figure 6:**
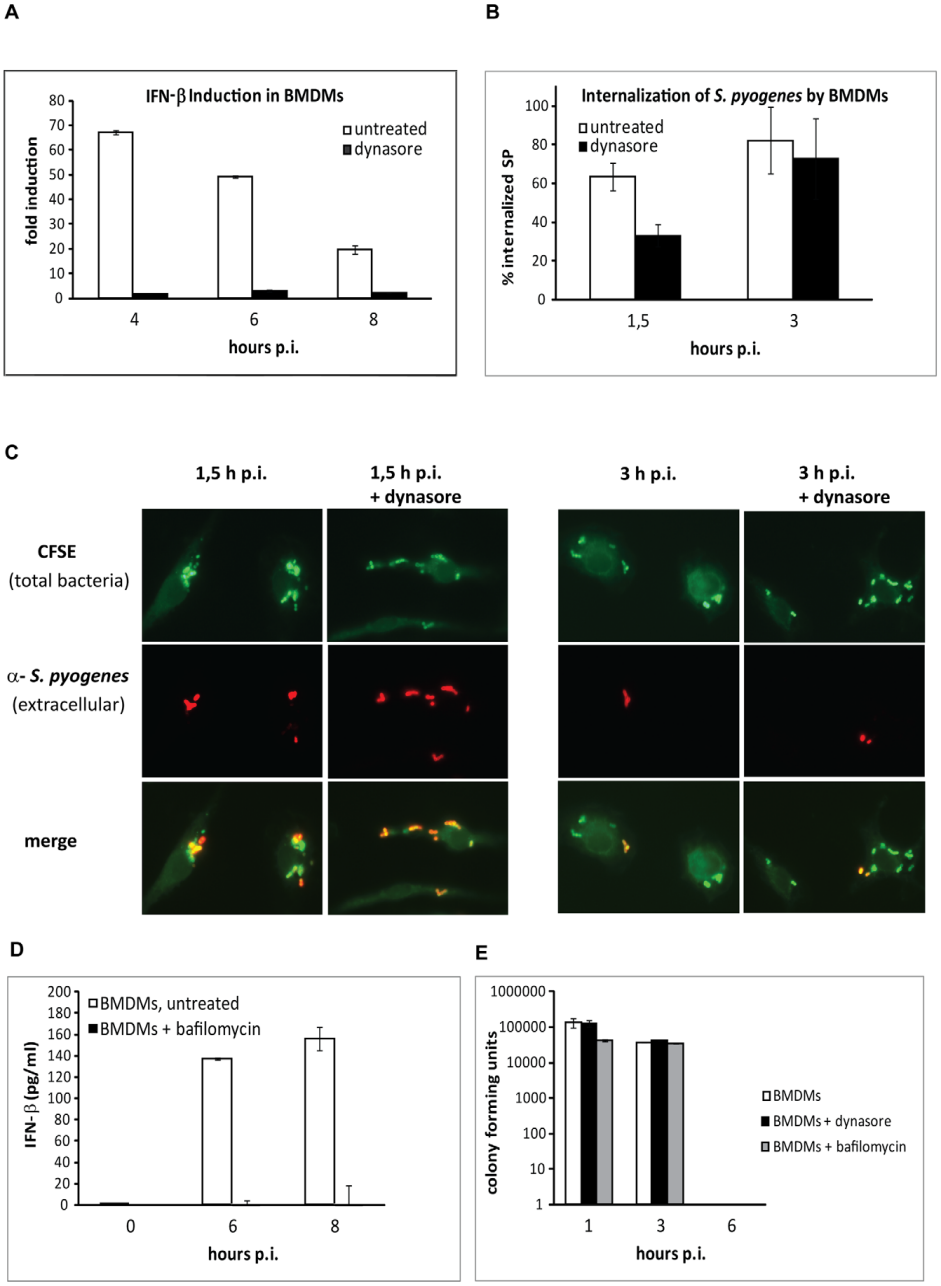
IFN-β production in response to *S. pyogenes* depends on phagosome maturation. (**A**) BMDMs pretreated (for 45 min) with dynasore or left untreated were infected with *S. pyogenes* (MOI = 100) for the indicated periods or left uninfected. At the indicated time points total RNA was isolated, reverse-transcribed and analyzed by qPCR for IFN-β. HPRT was used for normalization. Values represent mean ± SD; n = 3. (**B**) BMDMs pretreated (for 45 min) with dynasore or left untreated were infected with CFSE-labeled (green) *S. pyogenes* (MOI = 100) and fixed at indicated time points. Extracellular *S. pyogenes* were stained with anti-*S. pyogenes* antibody (red). The percentages of internalized *S. pyogenes* were calculated based on the number of red and green fluorescing bacteria in a field. At least five fields were counted and SDs were calculated. (**C**) Fluorescence microscopy imaging of cells treated as described in (B). Total *S. pyogenes* labeled with CFSE (green) and extracellular *S. pyogenes* stained post-fixation with anti-*S. pyogenes* antibody (red), as well as merge are shown. Green cells in the merge image represent internalized bacteria. Representative panels are shown. (**D**) BMDMs pretreated (for 45 min) with bafilomycin A1 or left untreated were infected with *S. pyogenes* (MOI = 100). At indicated time points, supernatants were collected and IFN-β release was measured as described in (A). Values represent mean ± SD; n = 3. (**E**) BMDMs treated with dynasore, bafilomycin A or left untreated were infected with *S. pyogenes* (MOI = 100). At indicated time points, serial dilutions of cellular lysates were plated to count colony-forming units (CFUs) that are displayed as log CFU.

It is becoming increasingly clear that the recognition of nucleic acids by innate immune cell receptors is not limited to viral infections but appears to be a more universal way of sensing danger signals which may originate from viruses, bacteria or even mammalian cells [10]. The response to nucleic acids by PRRs occurs either in the endosomal compartment or in the cytosol ensuring successful detection in both the phagosome and, in case of phagosomal escape of the pathogen, in the cytosol. We assumed that the *S. pyogenes*-derived PAMPs were recognized primarily during phagocytosis since *S. pyogenes* is not capable of further growth after internalization, although streptolysin O (SLO)-mediated escape from phagosome to autophagosome with a short-lived cytosolic intermediate occurs [36], [37], [38]. This assumption was supported by our findings that *S. pyogenes*-induced IFN-β was completely dependent on MyD88 in cDCs, while only to 50% in BMDMs (Fig. 2A, B), as the cytosolic PRRs do not signal via the MyD88 adaptor in these cells [10]. Furthermore, heat-killed *S. pyogenes*, which is assumed to lack the ability to deliver bacterial products into the cytosol [23], retained, albeit in BMDMs to only ∼50%, its capacity to induce IFN-β (Fig. S6A, B). Interestingly, heat-killed *S. pyogenes* was consistently a better IFN-β inducer than live bacteria in cDCs (Fig. S6B). To directly test the role of uptake of *S. pyogenes* in IFN-β induction, we treated BMDMs with dynasore prior to infection. Dynasore, an inhibitor of the small GTPase dynamin that is required for internalization of coated endosomal vesicles [39], is frequently used to assess the contribution of endosomal signaling to IFN-β production [40]. Dynasore completely abolished *S. pyogenes*-mediated induction of IFN-β (Fig. 6A). For IFN-β detection in the dynasore experiment, we assayed IFN-β mRNA rather than protein, since dynasore might interfere with protein secretion. To test whether dynasore inhibited uptake of *S. pyogenes,* we employed differential staining of extracellular an intracellular bacteria. *S. pyogenes* cells were first labeled with carboxyfluorescein succinimidyl ester (CFSE), followed by infection of dynasore- or untreated BMDMs. CFSE did not reduce the viability of *S. pyogenes* (data not shown). Infected cells were then fixed at 90 min and 180 min post infection, and the extracellular cell surface-bound bacteria were stained by anti-*S. pyogenes* antibodies. Ninety minutes after infection, less internalized bacteria were found in dynasore-treated cells, but 180 min after infection, the number of internalized bacteria was similar in dynasore-treated and untreated cells (Fig. 6B, C). It is unlikely that the inhibition of IFN-β by dynasore reflected only a delayed induction caused by the slower uptake since IFN-β induction was abolished by dynasore at any time point examined (up to 8 h, Fig. 6A). Thus, dynasore acted in steps following phagocytosis. Since dynamin, the target of dynasore, has been reported to affect maturation steps of phagocytic vesicles but not their formation [41] we examined the role of phagosomal maturation in IFN-β induction by employing bafilomycin A1, a specific inhibitor of the phagosomal H^+^ ATPase. Bafilomycin A1 blocked IFN-β production by *S. pyogenes*-infected BMDMs (Fig. 6D), suggesting that hydrolytic degradation of the pathogen was a critical event in IFN-β induction. However, the maturation of phagosomes was not needed for killing of bacteria, as demonstrated by comparable numbers of surviving bacteria in dynasore- or bafilomycin A1-treated and control cells (Fig. 6E). Similar effects of bafilomycin A1 were observed also in cDCs (data not shown), suggesting that the hydrolytic processing of internalized bacteria is fundamental for IFN-β induction in both BMDMs and cDCs.

### Endosomal delivery of *S. pyogenes*-derived RNA and DNA activate IFN-β production in cDCs and BMDMs, respectively

**Figure 7:**
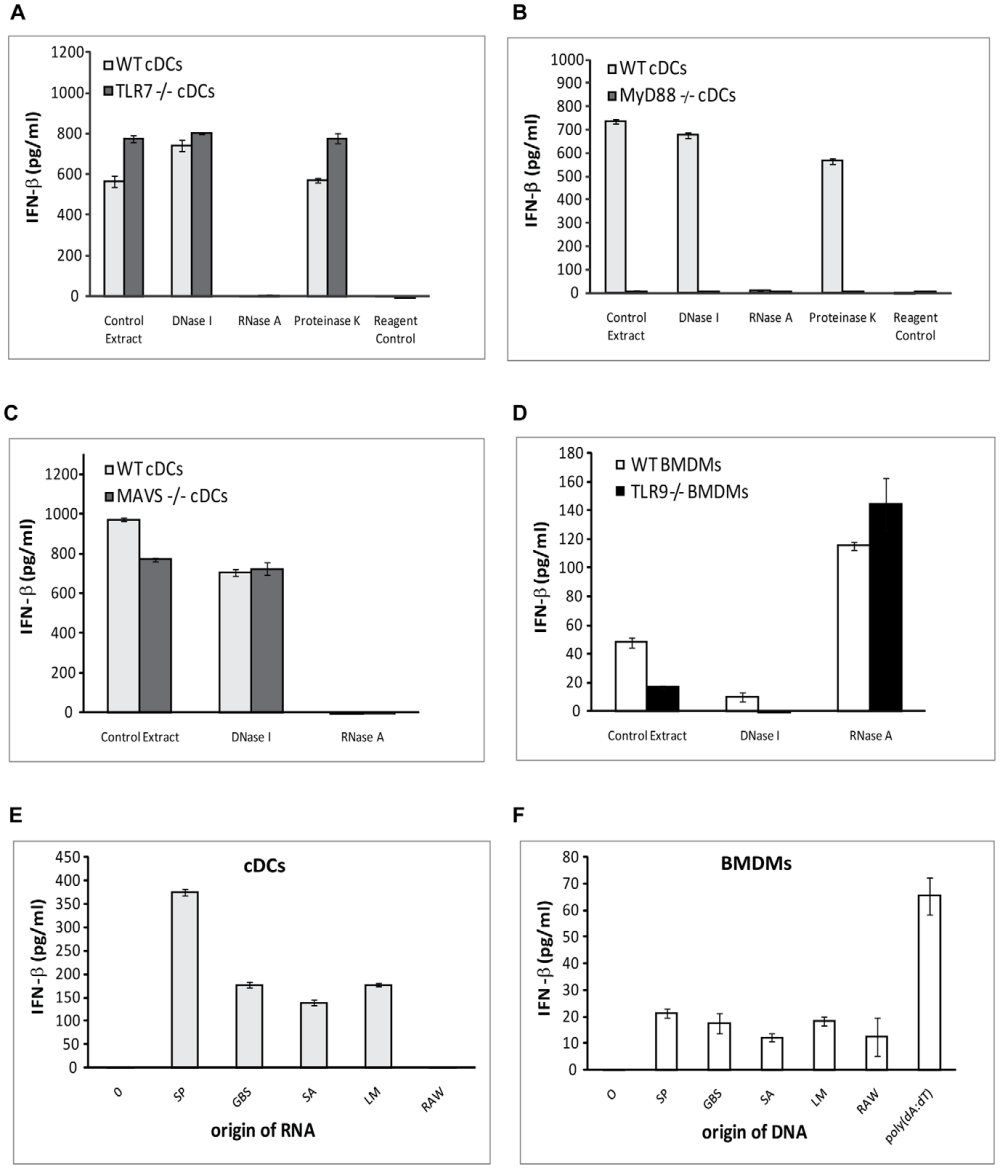
*S. pyogenes*-derived RNA and DNA delivered to endosomes induce IFN-β in cDCs and BMDMs, respectively. *S. pyogenes* cells were sonicated and the extracts were treated with either DNase I, RNase A, Proteinase K, or left untreated (control extract). The extracts and a reagent control were delivered into cDCs derived from TLR7^-/-^ (**A**), MyD88^-/-^ (**B**) and MAVS^-/-^ (**C**) or control (WT) mice using DOTAP. After stimulation for 6 h, supernatants were collected and IFN-β release was measured using ELISA. Values represent mean ± SD; n = 3. (**D**) BMDMs from TLR9^-/-^ and control (WT) mice were transfected with streptococcal extracts as described in (A). After 8 h supernatants were collected for IFN-β measurements by ELISA. (**E)** Purified RNA (5 µg) from *S. pyogenes* (SP), Group B streptococcus (GBS), *Staphylococcus aureus* (SA), *Listeria monocytogenes* (LM) and RAW 264.7 cells (RAW) were delivered into cDCs using DOTAP. After stimulation for 6 h supernatants were collected and IFN-β release was measured. Values represent mean ± SD; n = 3. (**F**) DNA (5 µg) from same organisms as in (E) as well as poly(dA:dT) were delivered into BMDMs and 8 h later IFN-β release was determined as in (E).

Microbial nucleic acids liberated during processing within the host cells serve often as danger signals. Accordingly, RNA from GBS was reported to induce IFN-β in a TLR7-dependent way in cDCs [25]. To test the role of *S. pyogenes*-derived nucleic acids in the induction of IFN-β, extracts of sonicated *S. pyogenes* treated with DNase I, RNase A, proteinase K or left untreated (control extract) were delivered into the cells using DOTAP. DOTAP is known to be taken up together with the bound cargo by the endocytic pathway [42]. *S. pyogenes*-derived complete extracts and the RNA-containing fraction where able to induce IFN-β in cDCs which was however not dependent on TLR7 (Fig. 7A). This was in agreement with the TLR7-independent IFN-β induction by live *S. pyogenes* (Fig. 4B) and supported our notion of distinct mode of IFN-β induction by *S. pyogenes* and GBS. To further substantiate the critical role of *S. pyogenes*-derived RNA in IFN-β induction in cDCs, we examined responses of MyD88^-/-^ cells to extracts from *S. pyogenes*. Responses of cDCs to the bacterial RNA-containing extracts were fully abolished in MyD88^-/-^ cells (Fig. 7B), which virtually mirrored the infection of cDCs with live *S. pyogenes* (Fig. 2B). Importantly, DOTAP-mediated delivery did not result in the activation of the cytosolic nucleic acid sensors NLRs, RLRs (RIG-I, MDA5 and LGP2), DAI or the recently reported LRRFIP1 since these PRRs signal independently of MyD88 [15], [34], [35], [43], [44]. To further support this notion we tested the adaptor MAVS (also called IPS-1, CARDIF, or VISA) that acts downstream of the RIG-I and MDA5 RNA sensors. cDCs derived from MAVS^-/-^ mice responded to *S. pyogenes*-derived RNA-containing extracts or to infection with live *S. pyogenes* by normal IFN-β induction (Fig. 7C and Fig. S7). Together, these results suggested that the *S. pyogenes* extracts administered using DOTAP represented a simplified yet still correct delivery of *S. pyogenes*-derived PAMPs to their authentic PRRs. We employed endosomal delivery to examine IFN-β induction by *S. pyogenes*-derived nucleic acids also in BMDMs. The experiment revealed that *S. pyogenes*-derived DNA but not RNA was required for IFN-β induction (Fig. 7D). The DNA-mediated IFN-β induction was even consistently increased after removal of RNA, which might be due a successful competition of the more abundant RNA for the DNA receptor. A concentration-dependent interference of deoxyribonucleic with ribonucleic acids with respect to TLR binding has been described [45]. Because of the involvement of DNA, we re-examined the function of TLR9. Treatment of TLR9^-/-^ BMDMs with *S. pyogenes* extracts revealed that TLR9 did not accomplish the recognition of *S. pyogenes*-derived DNA if RNA was removed (Fig. 7D). In contrast, the control extracts (i.e. containing both RNA and DNA) induced IFN-β in a TLR9-dependent way, suggesting that in the presence of RNA, the recognition of bacterial DNA is achieved by TLR9, whereas in the absence of RNA the recognition is shifted to a different PRR. Nevertheless, the overall contribution of TLR9 to *S. pyogenes*-induced IFN-β is negligible since TLR9^-/-^ BMDMs infected with live *S. pyogenes* produced comparable amounts of IFN-β (Fig. S3). Similar to induction by live *S. pyogenes* (Fig. 6A), the induction of IFN-β by *S. pyogenes*–derived DNA required a dynasore-sensitive step (Fig. S8).

Together, our data reveal that bacterial DNA and RNA are key players in induction of IFN-β by *S. pyogenes*-infected BMDMs and cDCs, respectively. Bacterial nucleic acids were in a number of studies reported to induce IFN-β although the mechanism is often not understood [46]. To directly compare the IFN-β-inducing properties of nucleic acids, we stimulated cDCs and BMDMs with purified RNA (for cDCs) or DNA (for BMDMs) from Gram-positive extracellular (GBS, *Staphylococcus aureus*), mostly extracellular (*S. pyogenes*) and intracellular (*Listeria monocytogenes*) bacteria and, for comparison, the RAW murine macrophages. cDCs displayed differential sensitivity to the tested RNA molecules. Whereas bacterial RNA robustly induced IFN-β, mammalian RNA failed to do so (Fig. 7E). In contrast, DNA of bacterial and mammalian origin were all capable of inducing IFN-β to similar levels which however remained 3-5 times lower than the levels activated by the known IFN-β inducer poly(dA:dT) (Fig. 7F). Consistently, linearized gel- and absorption column-purified pGEX plasmid DNA was also able to induce IFN-β (Fig. S9). None of the DNAs tested was able to induce TNF production suggesting that these DNA preparations did not contain significant amounts of other PAMPs (e.g. LTA) that could have provided a second stimulus potentially needed for IFN-β production (Fig. S10). Thus, in this simplified model of nucleic acid delivery, DNA was in general capable of IFN-β induction while the IFN-β-inducing properties of RNA were restricted to the bacterial molecules. It should be noted that, similar to infection with live *S. pyogenes*, IFN-β induction by the relevant nucleic acids was more robust in cDCs than in BMDMs, suggesting that cDCs might be the more important IFN-β producers *in vivo*.

## Discussion

Recognition of Gram-positive extracellular bacteria was long thought to be primarily driven by interactions between TLR2 and the bacteria-derived LTA or lipoproteins [47]. It was therefore surprising that the recognition of *S. pyogenes* and GBS occurred independently of TLR2 although both species produce TLR2 ligands [19], [20], [21], [48], [49]. Subsequent studies by us and others demonstrated that both *S. pyogenes* and GBS were also able to induce IFN-β in innate immune cells. Despite these similarities, *S. pyogenes* and GBS share only about 200 out of ∼2000 genes, and cause different diseases [50]. Although both pathogens have extracellular life cycles, without the ability of replication within host cells, *S. pyogenes* can invade and survive for several days in nonphagocytic cells [17], [18].

We provide evidence that type I IFN signaling is required for survival of mice challenged with *S. pyogenes* in a model of invasive subcutaneous infection. During the innate immune system-controlled phase of infection, type I IFNs regulate in multiple ways the host response. Type I IFNs can inhibit chemokine (e.g. CXCL1 and CXCL2) expression and thereby reduce recruitment of neutrophils to the site of infection in case of secondary pneumococcal pneumonia [51]. While the decreased neutrophil presence diminishes the local antibacterial activity, it also limits tissue damage and dissemination of bacteria. Consequently, both beneficial as well as detrimental effects of neutrophil infiltration were reported [51], [52]. Whether and how the higher neutrophil numbers and the more pronounced tissue destruction, which we have observed at the site of infection in the IFNAR1^-/-^ animals, contribute to the increased susceptibility to *S. pyogenes* will be investigated in future studies.

In bacterial infections, the type I IFN-producing cells are predominantly macrophages and cDCs, rather than plasmacytoid DCs [25], [53]. We therefore focused on macrophages and cDCs, the cell types required for successful defense of mice against *S. pyogenes* [8], [9]. We examined the production of IFN-β as the first type I IFN to be synthesized during infections [54]. BMDMs and cDCs displayed common as well as unique features with regard to the induction of IFN-β. In both cell types MyD88 fulfilled an important role, although in BMDMs about 50% of the generated IFN-β was independent of MyD88. In cDCs, the complete dependence on MyD88 was accompanied with a requirement for IRF5 and partially IRF1, which was consistent with the current view positioning these IRFs downstream of MyD88 [10]. This canonical MyD88/IRF axis was described to be triggered by TLR7 or TLR9 which we however ruled out as the *S. pyogenes*-sensing and IRF-activating TLRs in cDCs. The involvement of TLR8, the only IFN-β-inducing MyD88-dependent TLR not experimentally tested by us, is unlikely, since TLR8 possesses an inhibitory function in cytokine production [55]. The involvement of TLR11 and TLR12, the least understood MyD88-dependet TLRs, cannot be excluded although these PRRs were so far not associated with type I IFN induction. Thus, the identity of the PRR responsible for triggering the MyD88/IRF pathway in the response of cDCs to *S. pyogenes* remains to be determined.

In BMDMs, about half of the induced IFN-β was MyD88-independent yet it required IRF3, the IRF3 kinase TBK1 as well as the adaptor STING. TBK1/IRF3 can be activated by TLR3 and TLR4 via the adaptor TRIF rather than MyD88. Our data demonstrate that TRIF-deficient cells respond normally to *S. pyogenes* suggesting that TLR3 and TLR4 were not the upstream activators of the TBK1/IRF3 module. We confirmed this conclusion by the analysis of TLR3^-/-^ BMDMs. TLR4 was disqualified already in our previous study [19]. For the MyD88-dependent IFN-β production we could now also exclude the involvement of TLR7 and TLR9. Thus, both the MyD88-dependent and -independent IFN-β production is induced by *S. pyogenes* independently of TLRs. Since we tested the TLRs as well as the adaptors MyD88 and TRIF in single knockouts we cannot exclude a redundant use of these PRRs and adaptor molecules. TBK1/IRF3 can be activated by various cytosolic sensors [10]. The signaling by these cytosolic PRRs does not require MyD88, hence only the MyD88-independent part of IFN-β induction could potentially be explained by these molecules. Our experiments using BMDMs deficient in NOD1 and NOD2, two cytosolic PRRs recently shown to activate the TBK1/IRF pathway [32], [34], [35] and known to recognize peptidoglycan from both Gram-positive and –negative bacteria [56], ruled out a role of these PRRs in *S. pyogenes*-induced IFN-β induction.

The nature of the IFN-β-inducing bacterial product was tested by delivering complete *S. pyogenes* extracts or extracts depleted of DNA, RNA or protein to endosomes. The endosomal route was chosen since IFN-β production in *S. pyogenes*-infected cells was blocked by dynasore and bafilomycin A. Our observation that dynasore did not inhibit the uptake is consistent with reported function of the dynasore target dynamin in early pre-acidification steps of phagosome maturation, rather than in phagosome formation [41]. Thus, both dynasore and bafilomycin A are likely to have interfered with hydrolytic degradation of the internalized bacteria. This processing was not required for killing of bacteria since the survival of internalized *S. pyogenes* was not enhanced in cells treated with dynasore or bafilomycin A. This finding suggests that the initially formed immature phagosomes are already fully capable of killing *S. pyogenes*, and no further signaling-dependent maturation is required. The effect of dynasore and bafilomycin A could also be explained by the requirement of the PRRs for an environment restricted to mature phagosomes. Our data pointing at *S. pyogenes*-derived RNA as the principle IFN-β inducer in cDCs are consistent with a model wherein the RNA is liberated from the bacterial cells upon processing in phagosomes. The bacterial RNA is then sensed by an endosomal receptor that triggers the IFN-β-inducing MyD88/IRF pathway. This model is further supported by our findings that in cDCs signaling by *S. pyogenes* extracts was dependent on MyD88 thereby fully recapitulating signaling upon infection with live bacteria. Thus, we ruled out DOTAP-mediated misguiding of bacterial RNA to the cytosol where it could generate an artificial IFN-β response caused by the activation of cytosolic, i.e. MyD88-independent, PRRs. This was further confirmed by normal IFN-β response of cells deficient in MAVS, the adaptor downstream of the cytosolic RNA sensors RIG-I and MDA5. One reason for the involvement of MyD88 could be the need for a second, i.e. MyD88-dependent, signal in the IFN-β induction. The *S. pyogenes* extracts contained bacterial products (e.g. LTA) that could trigger MyD88. However, we observed similar IFN-β induction also by purified nucleic acids suggesting that a linear pathway was activated. Bacterial RNA as an inducer of IFN-β in cDCs has been reported for GBS [25]. However, GBS required TLR7 for sensing whereas *S. pyogenes* did not although both species employed MyD88. In BMDMs the IFN-β-inducing *S. pyogenes* extracts required the presence of bacterial DNA rather than RNA. TLR9-deficiency reduced detection of DNA in the complete extracts but not in extracts devoid of RNA. Thus, RNA appeared to channel bacterial DNA to TLR9. Alternatively, RNA might prevent DNA from escaping to the cytosolic PRR. Nevertheless, TLR9 does not play a decisive role in *S. pyogenes* sensing since IFN-β induction by live bacteria was independent of this PRR. This PRR signals via TBK1, STING and IRF3 in a partially MyD88-dependent way. Comparison with GBS-induced IFN-β by BMDMs reveals some parallels but also important differences [24]. Both pathogens signal via bacterial DNA and employ the TBK1/IRF3 pathway. For GBS it was proposed that the bacterial DNA escaped in a pore-forming-toxin-dependent way from the phagosomes into the cytosol of infected cells where it was sensed by an unknown IFN-β-inducing cytosolic PRR. However, the production of IFN-β was independent of MyD88 and required live bacteria, which is in marked difference to *S. pyogenes*. The proportion of IFN-β that was induced by *S. pyogenes* independently of MyD88 might be triggered by bacterial products liberated from the phagosome or during the short cytosolic presence of *S. pyogenes*. These products would then be accessible to cytosolic PRRs like RNA polymerase III, NLRs, RLRs, or DAI that all signal independently of MyD88 [34], [35], [43], [44], [57], [58]. We ruled out an exclusive sensing function of the recently identified cytosolic DNA sensor LRRFIP1 [15] since LRRFIP1 does not cause phosphorylation of IRF3, which is in contrast to our observation of *S. pyogenes*-induced IRF3 phosphorylation. To our knowledge the so far only reported cytosolic MyD88-dependent DNA sensors are the DHX9 and DHX36 helicases in plasmacytoid DCs [59]. Further studies are needed to clarify the role of these or related helicases in responses of BMDMs to *S. pyogenes*. Expectedly, the ability of *S. pyogenes*-derived DNA to induce IFN-β in BMDMs was not unique since other bacterial, mammalian or plasmid DNA molecules were also potent inducers. However, the IFN-β induction by RNA in cDCs was accomplished only by RNA from the tested Gram-positive bacteria but not mammalian cells. Thus, bacterial RNA must possess specific determinants recognized by host cells. In support of this, single-stranded RNA from Gram-positive bacteria but not Gram-negative bacteria was recently found to induce TNF in macrophages [60].

This study delineates the importance of type I IFN induction by innate immune cells for successful defense against *S. pyogenes*. In a similar infection model, MyD88 was recently found to be required for full blown immune response and survival [22]. Since our data reveal that MyD88 plays a critical role in the induction of IFN-β, it is conceivable that the decreased IFN-β production contributed to the high susceptibility of MyD88-deficient mice to the infection. Furthermore, comparison of BMDMs and cDCs revealed different mechanism of IFN-β induction. Both cell types employ unknown nucleic acid-sensing PRRs for IFN-β induction. It is striking that the PRRs required for the NF-κB -driven induction of TNF and IL-6 in *S. pyogenes*-infected BMDMs and cDCs have so far also not been identified [19], [20]. It appears that a successful recognition of *S. pyogenes* by the host and the full blown immune response strongly depend on functional phagocytosis and, consequently, on professional phagocytic cells. These cells and their ability to launch type I IFN production can prove important targets for therapeutical intervention.

## Materials and Methods

### Bacterial strains and culture

The *Streptococcus pyogenes* serotype M1 strain ISS 3348 (provided by Roberta Creti, Instituto Superiore di Sanita, Italy) was used for *in vitro* as well for *in vivo* experiments [61]. *S. pyogenes* was grown at 37°C with 5% CO_2_ without agitation in Todd-Hewitt-Broth (BD Biosciences) supplemented with 0.2% yeast extract and on trypcase soy agar containing 5% sheep blood (Biomerieux). The cell growth was turbidimetrically monitored at 620 nm with a microplate reader until mid-log phase was reached. For preparation of heat-killed *S. pyogenes*, bacteria were grown to mid-log phase, washed twice with sterile PBS and incubated for 20 min at 70°C in a water bath. *Streptococcus agalactiae* (Group B Streptococcus, GBS) NEM316 and *Listeria monocytogenes* LO28 were grown at 37°C in brain heart infusion broth (Difco). *Staphylococcus aureus* 25923 was grown at 37°C in Mueller-Hinton broth (Merck).

### Mice

IFNAR1^-/-^, MyD88^-/-^, IRF1^-/-^, IRF3^-/-^, TLR3^-/-^, TLR7^-/-^, TRIF^-/-^, TLR9^-/-^, IRF5^-/-^ and MAVS^-/-^ mice on C57BL/6 background were housed under specific pathogen-free conditions. C57BL/6 WT mice were purchased from Charles River Laboratories.

### Experimental model of *S. pyogenes* infection

Age- and sex-matched, pathogen free, 8–10 week old WT and IFNAR1^-/-^ mice (all C57BL/6) were used in all experiments. *S. pyogenes* strain ISS 3348 was grown for 4 hours to mid-logarithmic phase at 37°C using Todd-Hewitt-Broth (BD Biosciences), harvested by centrifugation at 6.500 rpm for 8 min, and washed twice in sterile isotonic saline. Bacteria were then resuspended at a concentration of 1×10^8^ CFU per 50 µl as determined by plating serial 10-fold dilutions on blood agar plates. Mice were lightly anesthetized by inhalation of isoflurane (Baxter) and the fur at the flank was partially removed by shaving. The inoculum was injected subcutaneously in one flank of each mouse. Survival of mice was monitored every 4–8 hours. Survival curves were analyzed by the Logrank Test in GraphPad Prism 4 (GraphPad software, San Diego, USA)**.** All data are presented as mean SD. Comparison between two groups was performed using t-test. P values ≤0.05 were considered as significant.

### Cell culture

Primary bone marrow derived macrophages (BMDMs) and conventional dendritic cells (cDCs) were obtained from the femur and tibia bone marrow of 6–10-week old mice (all C57BL/6). Macrophages were cultivated in Dulbeccós modified Eagle's medium (DMEM) supplemented with 10% fetal calf serum (GIBCO) in the presence of L929-cell derived CSF-1, as described [19]. Dendritic cells were cultivated for 7 days in DMEM supplemented with 10% fetal calf serum in the presence of X-6310 cell-derived GM-CSF as described [62].

### *S. pyogenes* infection *in vitro*

For infection assays, BMDMs and cDCs were seeded on 6-cm dishes (2×10^6^ cells/dish) in DMEM containing 10% FCS and L-cell-derived CSF-1 or X-6310 cell derived GM-CSF, respectively, without antibiotics. The cells were then infected as described previously [19]. In experiments with heat-killed *S. pyogenes* equal amounts of heat-killed and live bacteria (MOI = 100) were used for infection. Infections under conditions of blocked endocytosis were performed by pretreatment of cells with dynasore (Sigma-Aldrich, 8 µM) for 45 min prior to infection with *S. pyogenes*. For inhibition of phagosomal acidification, bafilomycin A1 (Santa Cruz, 1 µM) was added to cells 45 min prior to infection with *S. pyogenes*.

### Determination of colony forming units (CFUs)

2×10^5^ BMDMs and cDCs were seeded per well in a 24-well plate. Cells were left untreated or were pretreated with dynasore (8 µM) or bafilomycin A1 (1 µM) for 45 min before infection with *S. pyogenes* (MOI = 100). Infection was carried out as described above. 1 and 3 hours after infection, extracellular bacteria were removed by washing with sterile PBS and macrophages were lysed with 500 µl distilled water. Serial dilutions of cellular lysates were plated on TSA blood agar plates (Biomerieux) and the number of CFUs was determined by colony counting after growth for 24 hours at 37°C.

### Phagocytosis assays

The uptake of *S. pyogenes* was analyzed as described previously [63], [64]. BMDMs and cDCs of control (WT) or MyD88^-/-^ mice were seeded in 12-well-plates (5×10^5^ cells/ml). Cells were infected with CFSE-labeled *S. pyogenes* (MOI = 50 and MOI = 100) for 45 min at 37°C as described above. Phagocytosis was terminated by incubation at 4°C and addition of 1 ml ice-cold PBS. cDCs were then transferred to FACS-tubes and washed with PBS prior to treatment with proteinase K (1 ml/tube, 50 µg/ml, Promega) for 10 min at room temperature to remove surface-adherent *S. pyogenes*. Cells were washed twice with PBS and suspended in trypan blue-containing PBS (200 µg/ml) to quench remaining extracellular bacteria. Uptake was analyzed using a flow cytometer (Beckton Dickinson FACScalibur) and results are expressed as phagocytosis index, defined as the percentage of cells with internalized *S. pyogenes* multiplied by the mean fluorescent intensity. The same procedure was followed when using adherent BMDMs with the exception of incubating BMDMs with CFSE-labeled bacteria at 37°C and 4°C for 45 min. Non-phagocytosed bacteria were removed by washing adherent monolayer cells with PBS and surface-adherent *S. pyogenes* were digested using proteinase K. No trypan blue was used in case of BMDMs since quenching of extracellular bacteria was replaced by subtracting mean fluorescence multiplied by the percentage of positive cells at 4°C from mean fluorescence multiplied by percentage positive cells at 37°C. Statistical analysis was performed using the Graph Pad Prism 5 Program (Graph Pad Software, San Diego, USA). Differences between WT and MyD88^-/-^ mice were analyzed by one-way ANOVA followed by Dunn's post test. Criteria for significance were p<0.05.

### Generation and transfection of *S. pyogenes* extracts

*S. pyogenes* was grown to OD_620_ = 0.3 and 5 ml aliquots of the bacterial suspensions were spun at 6.500 rpm for 8 min. The bacteria were resuspended in 400 µl sterile PBS followed by disruption using a Bandelin Sonopuls GM70 sonicator for 3×2 min. Remaining debris was removed by centrifugation at 13.200 rpm for 15 min at 4°C. The supernatants were pooled, adjusted to contain 2 mM MgCl_2_, 50 mM KCl and 20mM Tris-HCl (pH 8) and divided into four aliquots. The same was done with sterile PBS for the reagent control. Remaining extract was frozen at −20°C for later usage. Aliquots were digested with DNase I (Roche, 100 U/ml), RNase A (Roche, 333 µg/ml) or proteinase K (Sigma-Aldrich, 1 mg/ml) or left-untreated (control extract). Extracts treated with either DNase I or RNase A were incubated at 37°C for 45 min, extracts digested with proteinase K were incubated for 1 hour at 37°C. After incubation, EGTA for proteinase K (final 2 mM) treatment or EDTA (pH 8, final 2.5 mM) for DNase I and RNase A treatment were added to extracts. In case of the reagent control and control extract, both EGTA and EDTA were used. The extracts were then incubated at 70°C for 10 min and centrifuged for 5 min at 13.200 rpm at 4°C. Supernatants were collected and subsequently frozen at −20°C until use. 10 µl of these extracts (containing 5 µg of nucleic acids) were then allowed to form complexes with 30 µl DOTAP (Roche). Transfections were carried out in 6-cm dishes containing 2×10^6^ BMDMs or cDCs in 1 ml/dish, according to the manufactureŕs instructions.

### DNA and RNA preparation from bacteria and transfection of cells

Isolation of bacterial DNA and RNA was performed according to standard techniques, with some modifications [65]. Briefly, for RNA isolation bacteria were grown to mid-log phase and lysed using 2% SDS and 1 mg/ml proteinase K (Sigma-Aldrich). For isolation of RNA, Trizol LS reagent (Invitrogen) was used. Afterwards, RNA was treated with DNase using TURBO DNA-free (Ambion). For DNA preparation, over-night cultures of bacteria were harvested, resuspended in TE buffer (10 mM Tris-HCl pH 8, 1 mM EDTA) and treated with lysozyme (Sigma, 2,5 mg/ml) for 30 min at 37°C. Then, heat-inactivated RNase A (Roche, 10 mg/ml), 20% SDS and 0,5 M EDTA were added. After incubation for 40 min at 37°C, 20 mg/ml Proteinase K was added and further incubated for 30 min at 37°C. DNA was isolated using Phenol:Chloroform:Isoamylalcohol (Sigma-Aldrich). After isolation, bacterial DNA was treated with RNase A (10 mg/ml) for 30 min at 37°C. BMDMs or cDCs (2×10^6^ cells per 6-cm dish) were seeded 24 h prior to transfection with 5 µg DNA or RNA using DOTAP as described for transfection of bacterial extracts. The transfection mixture was added to dishes containing 1 ml (for BMDMs) or 3 ml (for cDCs) medium.

### Isolation of DNA and RNA from mammalian cells

For isolation of DNA, RAW 267.4 cells were lysed in direct PCR tail lysis buffer (Peqlab) at 55°C over night. DNA was then isolated using Phenol:Chloroform:Isoamylalcohol (Sigma-Aldrich). After isolation, DNA was treated with heat-inactivated RNase A (10 mg/ml) for 30 min at 37°C. RNA was isolated using Trizol LS reagent (Invitrogen) and treated with DNase using TURBO DNA-free (Ambion).

### Histology

The pyogenic lesions or control flanks (PBS-injected) of infected IFNAR1^-/-^ and WT mice were removed 48 hours after *S. pyogenes* injection. The tissue samples were fixed in 4% formalin and subsequently embedded in paraffin. 4 µm sections were stained with hematoxylin-eosin. Immunohistochemical staining of granulocytes was performed as described previously [66]. Briefly, the sections were deparaffinized, rehydrated and digested by a solution of pepsin 0.25% (Sigma-Aldrich) in 0.01; HCL. After being rinsed the slides were exposed to FITC-labeled rat anti-mouse Ly6G antibody (BD Biosciences) or corresponding isotype control IgG (Emfret Analytics, Germany) followed by a further incubation with rabbit anti-FITC Ab (Zymed, Invitrogen) in normal mouse serum. Finally the sections were incubated with polyclonal anti-rabbit-HRP Ab (Immunologic, The Netherlands) and visualized using 3,3-diaminobenzidine-tetrahydrochloride (Vector Laboratories, Burlingame, CA). Counterstaining was done with hemalaun solution.

### Fluorescence microscopy

BMDMs were seeded on glass cover slips (5×10^5^ cells/3,5 cm dish) in DMEM containing 10% FCS and L-cell-derived CSF-1, without antibiotics. Cells were infected with CFSE-labeled *S. pyogenes* (MOI = 100) as described above. Infections were performed after pretreatment or mock-treatment of cells with dynasore (Sigma-Aldrich, 8 µM) for 45 min prior to infection. At the indicated time points, cells were fixed with 1% formaldehyde, blocked with 1% BSA in PBS and incubated with primary anti-*S. pyogenes* rabbit antibody (kind gift from I. Julkunen), followed by Alexa Fluor 594 goat anti-rabbit IgG (Invitrogen). Cover slips were mounted on microscope slides with Fluorescent mounting medium (Dako). To quantify the ratio of internalized *S. pyogenes*, green and red fluorescent bacteria were counted under GFP and rhodamine filters, respectively. The percentage of internalized bacteria was calculated by subtracting the number of external bacteria (red) from the total number of CFSE-labeled bacteria (green), then dividing by the number of CFSE-labeled bacteria.

### Cytokine measurement

IFN-β in supernatants of infected macrophages and dendritic cells was measured by ELISA using the VeriKine Mouse Interferon Beta Kit (PBL Biomedical Laboratories), according to the manufactureŕs instructions. Samples were applied undiluted. TNF in supernatants of infected macrophages were assayed using a DuoSET ELISA kit (R&D Systems). Samples were diluted 1:2 in reagent diluent.

### Antibodies

Antibodies to Tyr701-phosphorylated Stat1 (pY701-S1), phospho-IRF-3 (Ser396) and TBK1 were purchased from Cell Signalling. The antibody against p38 MAPK was from Santa Cruz Biotechnology, the antibody against IRF3 was from Zymed (Invitrogen). The antibody to Stat1-C-terminus was previously described [67].

### Western blot analysis

After treatment, whole cell extracts were prepared and assayed by Western blotting as described previously [67]. Detection and quantification of signals were performed using the infrared imaging system Odyssey (LI-COR Biosciences) using fluorophore-linked secondary antibodies (Molecular Probes and Rockland).

### Quantitative RT-PCR (qRT-PCR)

Total RNA was isolated using Trizol LS reagent (Invitrogen). Reverse transcription of total RNA was performed using oligo (dT)_18_ as primer and Mu-MLV reverse transcriptase (Fermentas) and the cDNA was subsequently used to amplify SOCS1 by qRT-PCR. qRT-PCR was run on a Realplex Mastercycler (Eppendorf). For SOCS1 and STING Quantitect Primer Assays (QIAGEN) were used. For IFN-β, IFN-beta-fwd 5′- TCAGAATGAGTGGTGGTTGC -3′ and IFN-beta-rev 5′ GACCTTTCAAATGCAGTAGATTCA 3′ primers were used. For HPRT, the housekeeping gene for normalization, the following primers were used: HPRT-fwd 5′-GGATTTGAATCACGTTTGTGTCAT-3′, and HPRT-rev 5′-ACACCTGCTAATTTTACTGGCAA-3′. Amplification of DNA was monitored by SYBR Green (Molecular Probes) [68].

### siRNA–mediated silencing

BMDMs were seeded on 6-cm dishes (2×10^6^ cells) in DMEM containing 10% FCS and L-cell-derived CSF-1 without antibiotics. Murine TBK1 and STING (ON-TARGETplus, Dharmacon) siRNA (60 pmol), or a non-target control-siRNA were mixed with 1 ml Opti-MEM I reduced serum medium (GIBCO) and transfected into BMDMs using 10 µl Lipofectamine RNAiMAX (Invitrogen) according to the manufactureŕs instructions. 48 hrs after transfection, medium was replaced by medium without siRNA and antibiotics. After additional 24 hours cells were infected with *S. pyogenes* (MOI = 100).

### Ethics statement

All animal experiments were discussed and approved by the Animal Care and Use Committee of the Medical University of Vienna, and carried out in accordance with the Austrian law for animal care (GZ 680 205/67-BrGt/2003). Animal experiments were authorized through the permission BMWF-66.009/0031-II/10b/2008 issued by the Austrian Ministry of Science to PK and SK.

### List of accession numbers in GenBank

IL6: NM_031168, MyD88: NM_010851, TLR2: NM_011905, TLR4: NM_021297, TLR7: NM_133211, TBK1: NM_019786, STING: NM_028261, MAVS: NM_144888, TRIF: NM_174989, IRF1: NM_008390, IRF3: NM_016849, IRF5: NM_012057, TLR3: NM_126166, TLR9: NM_031178, IFNAR1: NM_010508, NOD1: NM_172729, NOD2: NM_145857, HPRT: NM_013556.

## Supporting Information

Figure S1 cDCs produce higher amounts of IFN-β in response to *S. pyogenes* than BMDMs. Both cell types respond in MOI-dependent way. (A) BMDMs and cDCs were left untreated or infected with *S. pyogenes* (MOI = 100). At indicated time points supernatants were collected and IFN-β release was measured. Mean values ± SD (n = 3) are shown. (B) BMDMs and cDCs were left untreated or infected with *S. pyogenes* at MOI = 20, MOI = 50 or MOI = 100. Supernatants were collected after 6 h (cDCs) or 8 h (BMDMs) and IFN-β release was measured as in (A).
(0.17 MB TIF)

Figure S2 MyD88 deficiency results in stronger Stat1 activation and reduced SOCS1 expression in response to *S. pyogenes*. Control (WT) and MyD88^-/-^ BMDMs were infected with *S. pyogenes* (MOI 100) or left untreated. At indicated time points, either whole cell extracts were prepared or total mRNA was extracted. (A) Stat1 activation was determined by Western blotting using antibody to phosphorylated Stat1 (pY-S1). For loading control, the membrane was reprobed using antibodies to total Stat1 (Stat1) and p38MAPK (p38). Note the double band on the pY-S1 blot represents the phosphorylated forms of both Stat1 splicing isoforms Stat1-α and Stat1-β. Loading control (Stat1) was performed with an antibody directed to the C-terminus of Stat1, which is absent in the Stat1-β isoform. (B) total RNA was reverse-transcribed and analyzed by qPCR for SOCS1 expression after normalization to HPRT. These data represent one of at least three independent infection experiments with different mice from each genotype.
(0.32 MB TIF)

Figure S3 TLR9 is not required for IFN-β induction by *S. pyogenes*. Control (WT) and TLR9-/- BMDMs were infected with S. pyogenes (MOI 100) and supernatants were collected at indicated time points. IFN-β release was measured in three independent infection experiments. Values represent mean ± SD; n = 3.
(0.12 MB TIF)

Figure S4 Silencing efficiency of STING expression. BMDMs were transfected with siRNA specific for STING or non-target control siRNA. siRNA-treated cells were then infected with *S. pyogenes* (MOI = 100) or left uninfected (as described in Fig. 4F). At indicated time-points, total RNA was extracted, reverse transcribed and analyzed by qPCR for STING expression after normalization to HPRT. These data represent one of at least three independent infection experiments. Mean values ± SD are shown (n = 3).
(0.17 MB TIF)

Figure S5 NOD1 and NOD2 are not required for IFN-β induction by *S. pyogenes*. BMDMs from control (WT), NOD1^-/-^ or NOD2^-/-^ mice were infected with *S. pyogenes* (MOI = 100). Whole cell extracts were prepared and supernatants were collected and at indicated time points. (A) Stat1 activation was determined by Western blotting using an antibody to phosphorylated Stat1 (pY-S1). Antibody to total Stat1 was used for loading control. (B) IFN-β release after 6 h of infection was measured in three independent infection experiments. Values represent mean ± SD; n = 3.
(0.24 MB TIF)

Figure S6 Heat-killed *S. pyogenes* causes induction of IFN-β in BMDMs and cDCs. BMDMs (A) and cDCs (B) were infected with equal amounts of live and heat-killed *S. pyogenes* (MOI 100) or left untreated. After the indicated time, supernatants were collected and IFN-β release was measured using ELISA. Mean ± SD; n = 3.
(0.17 MB TIF)

Figure S7 The adaptor MAVS is not needed for IFN-β induction by *S. pyogenes* in cDCs. cDCs from control (WT) and MAVS^-/-^ mice were infected with *S. pyogenes* (MOI 100). After 4 and 6 h, supernatants were collected and IFN-β release was measured using ELISA. Mean ± SD; n = 3.
(0.12 MB TIF)

Figure S8 Dynasore inhibits IFN-β production induced by extracts derived from *S. pyogenes*. BMDMs were pretreated (for 45 min) with dynasore or left untreated. *S. pyogenes* cells were sonicated and the extracts were treated with either DNase I, RNase A, Proteinase K, or left untreated (control extract). These extracts were delivered into BMDMs using DOTAP. After stimulation for 8 h, supernatants were collected and IFN-β release was measured using ELISA. Values represent mean ± SD; n = 3.
(0.11 MB TIF)

Figure S9 Plasmid DNA induces IFN-β production in BMDMs. Plasmid pGEX was linearized by digestion with EcoRI, gel-purified and eluted from DNA purification column. Five µg of the linearized and purified pGEX DNA or *S. pyogenes* (SP)-derived DNA were transfected into BMDMs using DOTAP. Supernatants were collected 8 h later and IFN-β release was determined. Values represent mean ± SD; n = 3.
(0.11 MB TIF)

Figure S10 DNA from Gram-positive bacteria does not induce TNF after transfection into BMDMs. Purified DNA (5 µg/ml) from *S. pyogenes* (SP), Group B streptococcus (GBS), *Staphylococcus aureus* (SA), *Listeria monocytogenes* (LM), RAW 264.7 cells (RAW) and poly(dA:dT) was delivered into BMDMs using DOTAP. After stimulation for 8 h supernatants were collected and TNF release was measured. Values represent mean ± SD; n = 3.
(0.12 MB TIF)
