# Supplementary figures and images for: Type I Interferon Production Induced by Streptococcus pyogenes-Derived Nucleic Acids Is Required for Host Protection
Source: PLoS Pathog. 2011 May 19;7(5):e1001345. doi: 10.1371/journal.ppat.1001345 (PMC3098218; doi:10.1371/journal.ppat.1001345)

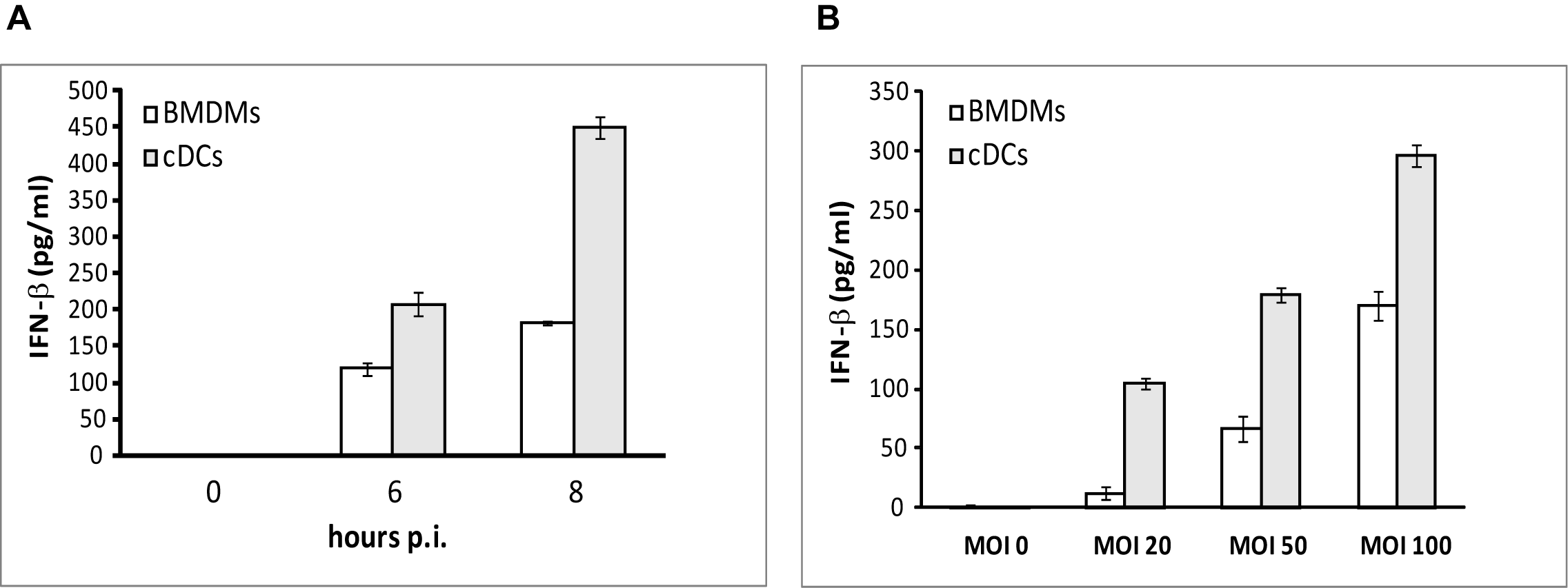

Supplement: Figure S1 — cDCs produce higher amounts of IFN-β in response to S. pyogenes than BMDMs. Both cell types respond in MOI-dependent way. (A) BMDMs and cDCs were left untreated or infected with S. pyogenes (MOI = 100). At indicated time points supernatants were collected and IFN-β release was measured. Mean values ± SD (n = 3) are shown. (B) BMDMs and cDCs were left untreated or infected with S. pyogenes at MOI = 20, MOI = 50 or MOI = 100. Supernatants were collected after 6 h (cDCs) or 8 h (BMDMs) and IFN-β release was measured as in (A). (0.17 MB TIF) [file ppat.1001345.s001.tif]

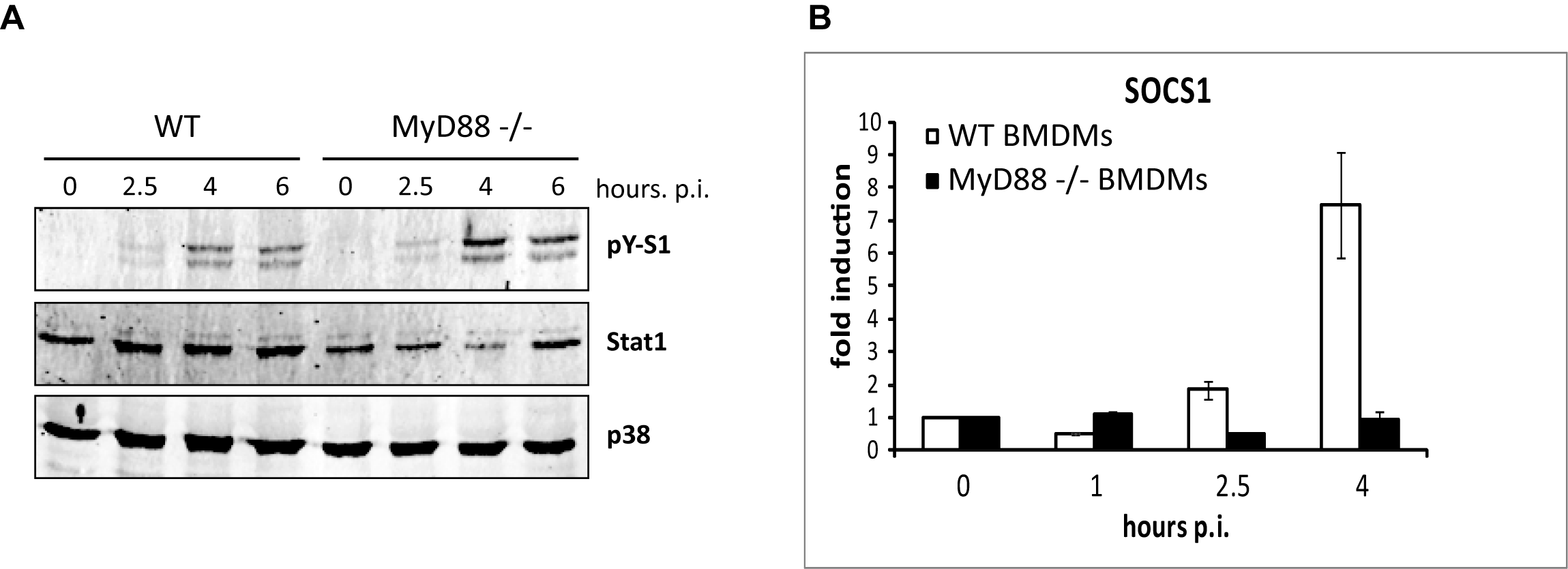

Supplement: Figure S2 — MyD88 deficiency results in stronger Stat1 activation and reduced SOCS1 expression in response to S. pyogenes. Control (WT) and MyD88-/- BMDMs were infected with S. pyogenes (MOI 100) or left untreated. At indicated time points, either whole cell extracts were prepared or total mRNA was extracted. (A) Stat1 activation was determined by Western blotting using antibody to phosphorylated Stat1 (pY-S1). For loading control, the membrane was reprobed using antibodies to total Stat1 (Stat1) and p38MAPK (p38). Note the double band on the pY-S1 blot represents the phosphorylated forms of both Stat1 splicing isoforms Stat1-α and Stat1-β. Loading control (Stat1) was performed with an antibody directed to the C-terminus of Stat1, which is absent in the Stat1-β isoform. (B) total RNA was reverse-transcribed and analyzed by qPCR for SOCS1 expression after normalization to HPRT. These data represent one of at least three independent infection experiments with different mice from each genotype. (0.32 MB TIF) [file ppat.1001345.s002.tif]

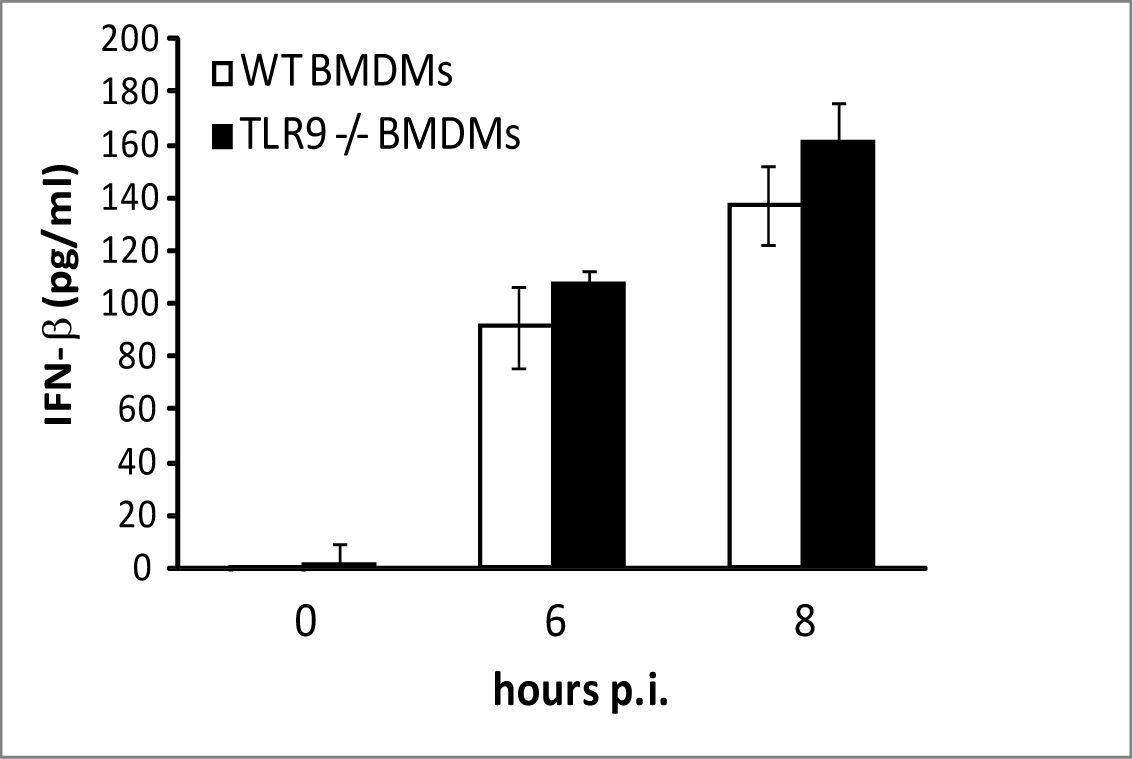

Supplement: Figure S3 — TLR9 is not required for IFN-β induction by S. pyogenes. Control (WT) and TLR9-/- BMDMs were infected with S. pyogenes (MOI 100) and supernatants were collected at indicated time points. IFN-β release was measured in three independent infection experiments. Values represent mean ± SD; n = 3. (0.12 MB TIF) [file ppat.1001345.s003.tif]

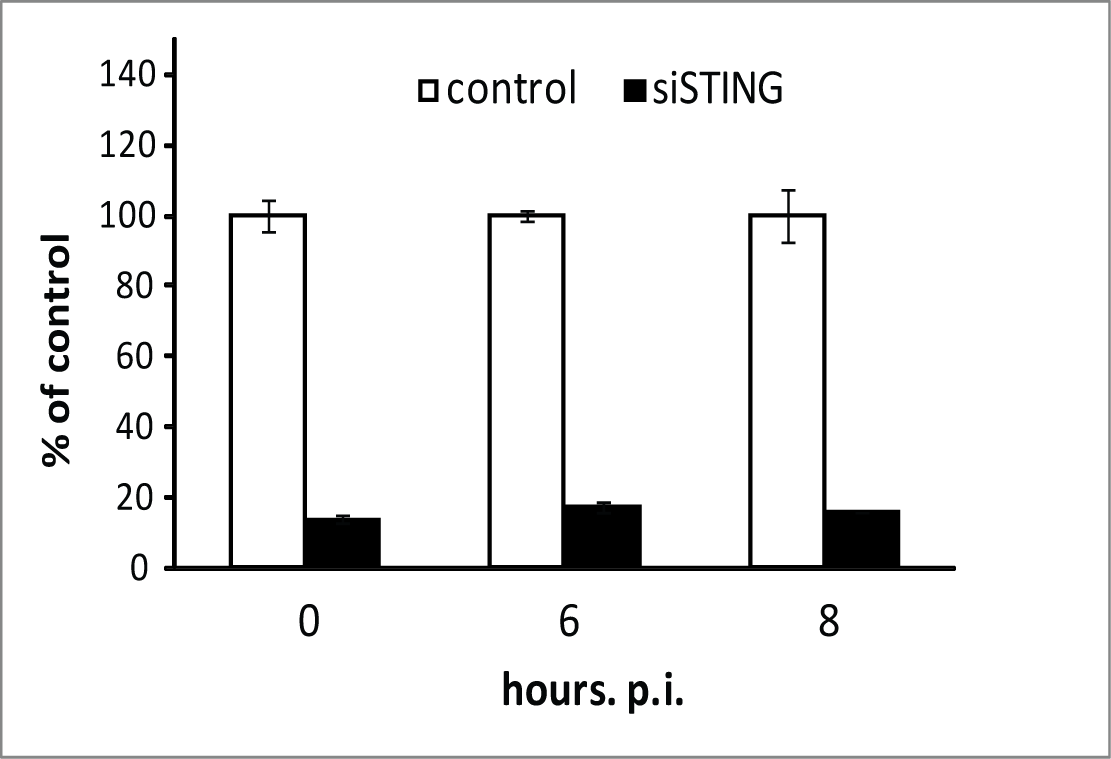

Supplement: Figure S4 — Silencing efficiency of STING expression. BMDMs were transfected with siRNA specific for STING or non-target control siRNA. siRNA-treated cells were then infected with S. pyogenes (MOI = 100) or left uninfected (as described in Fig. 4F). At indicated time-points, total RNA was extracted, reverse transcribed and analyzed by qPCR for STING expression after normalization to HPRT. These data represent one of at least three independent infection experiments. Mean values ± SD are shown (n = 3). (0.17 MB TIF) [file ppat.1001345.s004.tif]

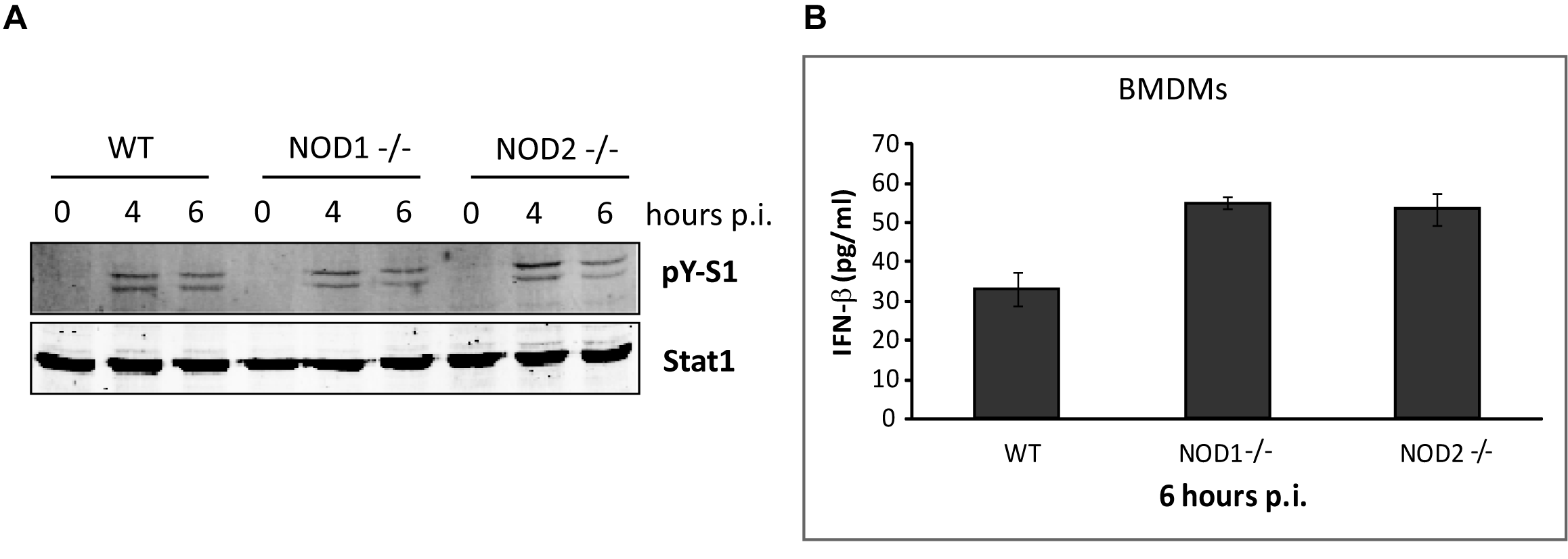

Supplement: Figure S5 — NOD1 and NOD2 are not required for IFN-β induction by S. pyogenes. BMDMs from control (WT), NOD1-/- or NOD2-/- mice were infected with S. pyogenes (MOI = 100). Whole cell extracts were prepared and supernatants were collected and at indicated time points. (A) Stat1 activation was determined by Western blotting using an antibody to phosphorylated Stat1 (pY-S1). Antibody to total Stat1 was used for loading control. (B) IFN-β release after 6 h of infection was measured in three independent infection experiments. Values represent mean ± SD; n = 3. (0.24 MB TIF) [file ppat.1001345.s005.tif]

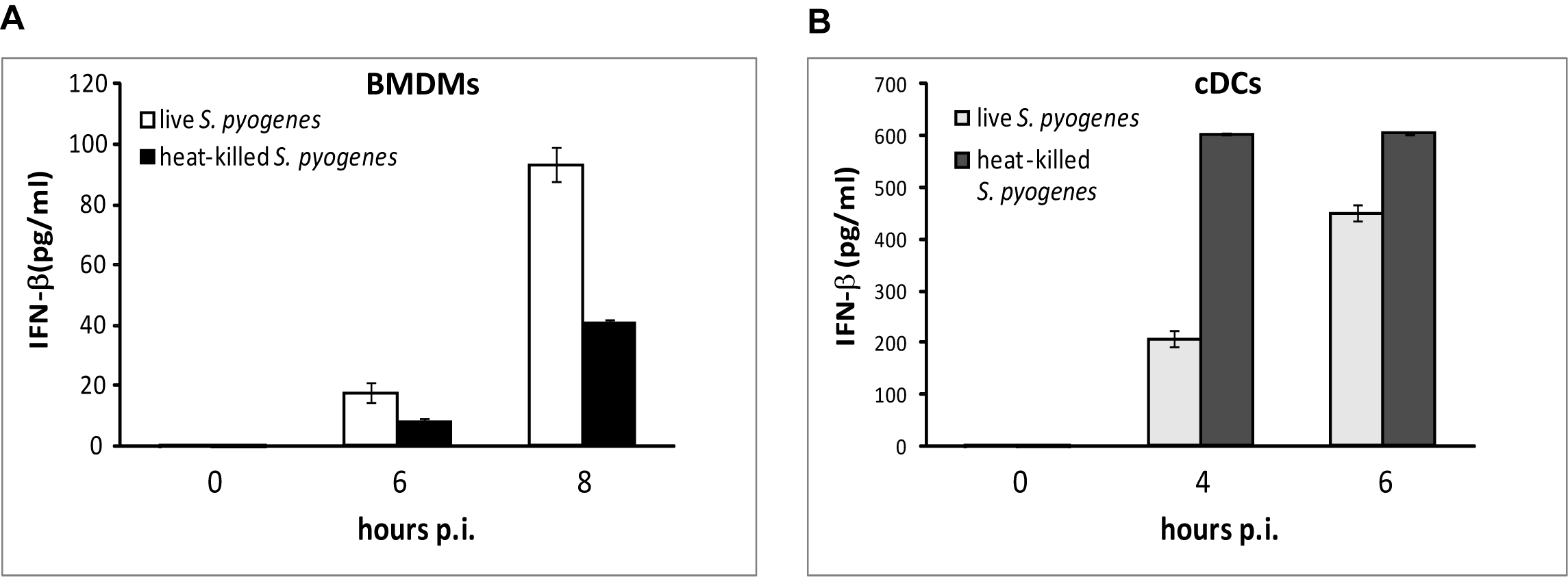

Supplement: Figure S6 — Heat-killed S. pyogenes causes induction of IFN-β in BMDMs and cDCs. BMDMs (A) and cDCs (B) were infected with equal amounts of live and heat-killed S. pyogenes (MOI 100) or left untreated. After the indicated time, supernatants were collected and IFN-β release was measured using ELISA. Mean ± SD; n = 3. (0.17 MB TIF) [file ppat.1001345.s006.tif]

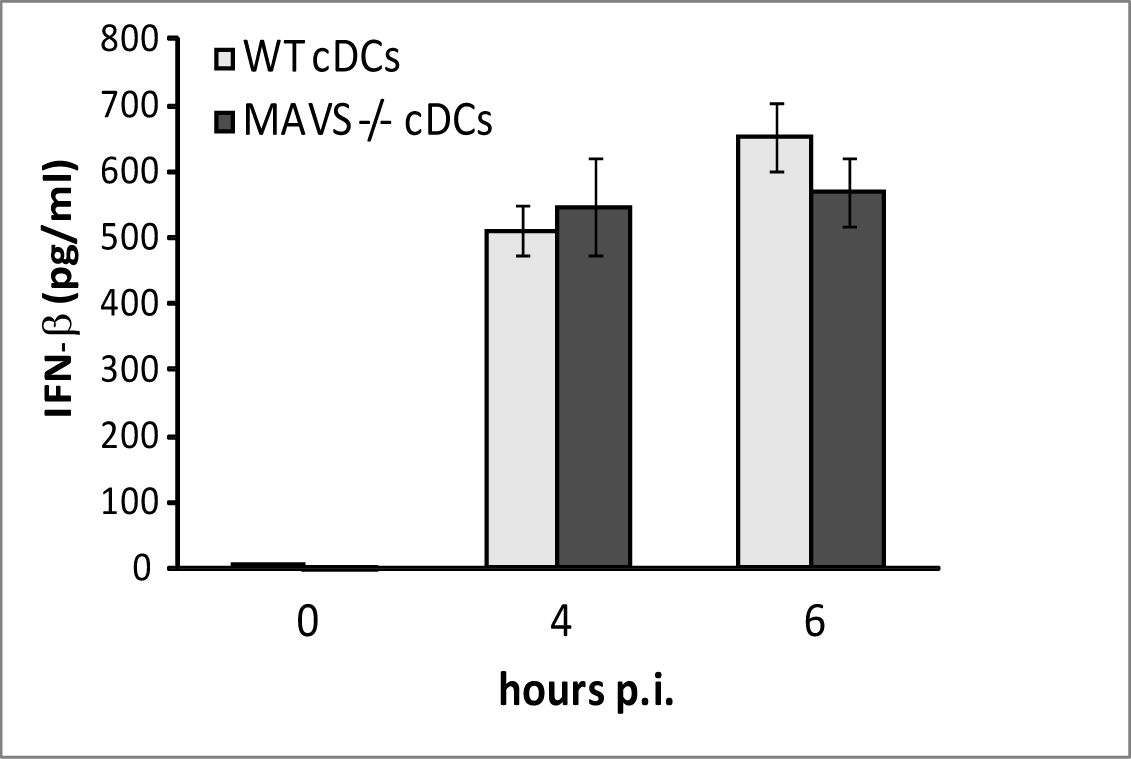

Supplement: Figure S7 — The adaptor MAVS is not needed for IFN-β induction by S. pyogenes in cDCs. cDCs from control (WT) and MAVS-/- mice were infected with S. pyogenes (MOI 100). After 4 and 6 h, supernatants were collected and IFN-β release was measured using ELISA. Mean ± SD; n = 3. (0.12 MB TIF) [file ppat.1001345.s007.tif]

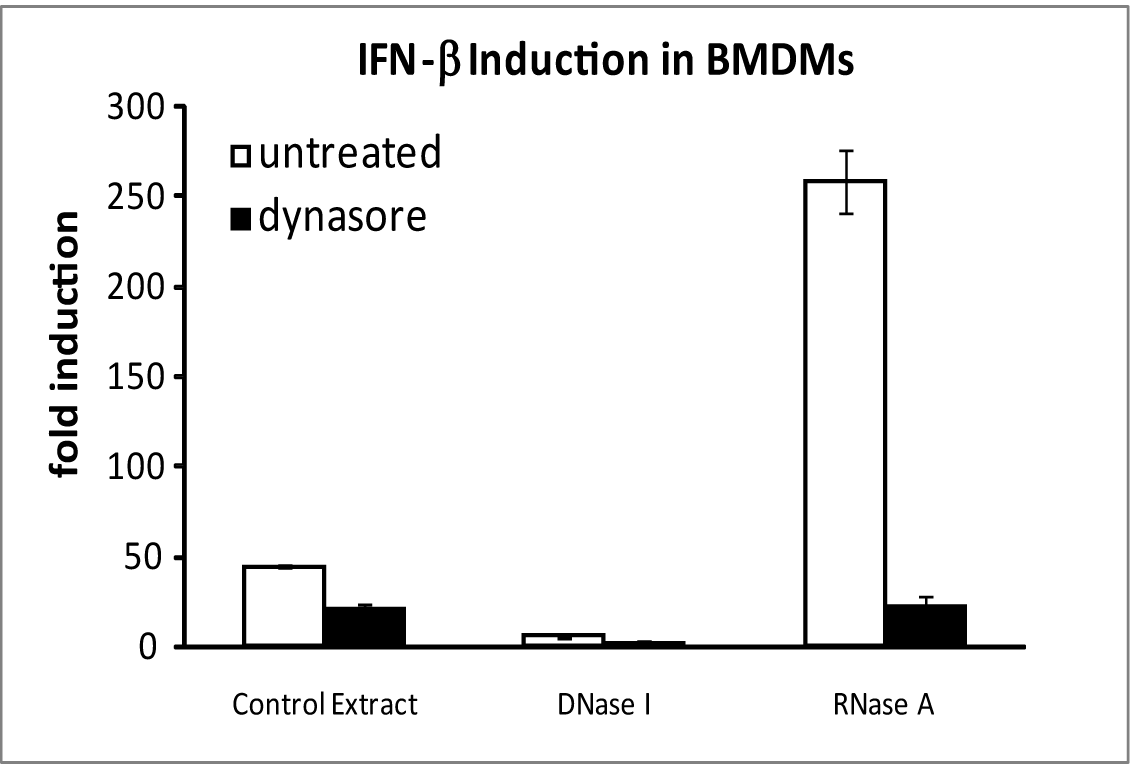

Supplement: Figure S8 — Dynasore inhibits IFN-β production induced by extracts derived from S. pyogenes. BMDMs were pretreated (for 45 min) with dynasore or left untreated. S. pyogenes cells were sonicated and the extracts were treated with either DNase I, RNase A, Proteinase K, or left untreated (control extract). These extracts were delivered into BMDMs using DOTAP. After stimulation for 8 h, supernatants were collected and IFN-β release was measured using ELISA. Values represent mean ± SD; n = 3. (0.11 MB TIF) [file ppat.1001345.s008.tif]

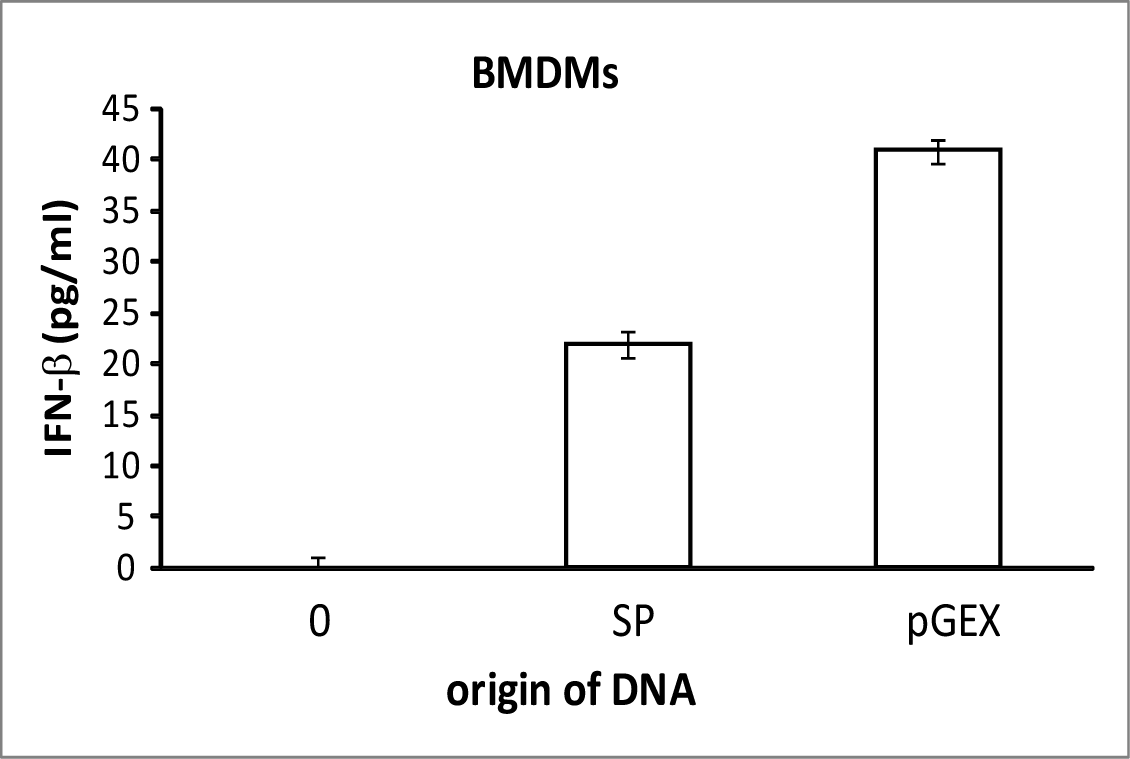

Supplement: Figure S9 — Plasmid DNA induces IFN-β production in BMDMs. Plasmid pGEX was linearized by digestion with EcoRI, gel-purified and eluted from DNA purification column. Five µg of the linearized and purified pGEX DNA or S. pyogenes (SP)-derived DNA were transfected into BMDMs using DOTAP. Supernatants were collected 8 h later and IFN-β release was determined. Values represent mean ± SD; n = 3. (0.11 MB TIF) [file ppat.1001345.s009.tif]

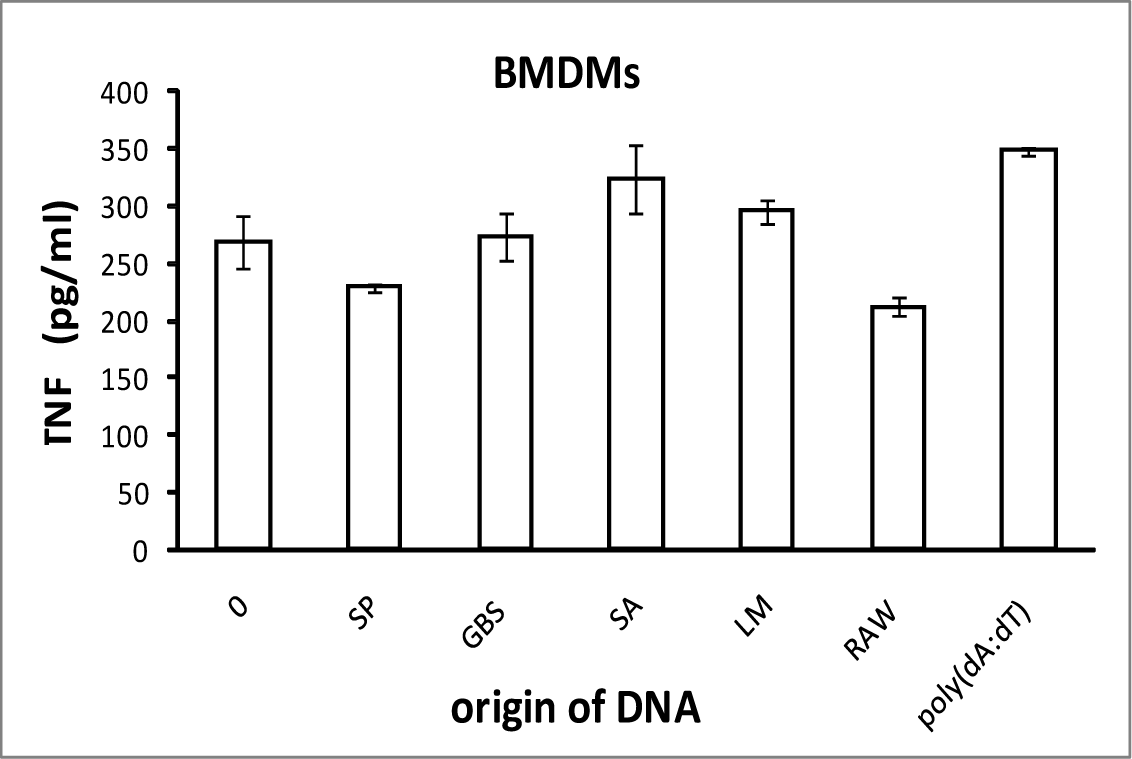

Supplement: Figure S10 — DNA from Gram-positive bacteria does not induce TNF after transfection into BMDMs. Purified DNA (5 µg/ml) from S. pyogenes (SP), Group B streptococcus (GBS), Staphylococcus aureus (SA), Listeria monocytogenes (LM), RAW 264.7 cells (RAW) and poly(dA:dT) was delivered into BMDMs using DOTAP. After stimulation for 8 h supernatants were collected and TNF release was measured. Values represent mean ± SD; n = 3. (0.12 MB TIF) [file ppat.1001345.s010.tif]
